# Training primary health care providers in Colombia, Mexico and Peru to increase alcohol screening: Mixed-methods process evaluation of implementation strategy

**DOI:** 10.1177/26334895221112693

**Published:** 2022-07-15

**Authors:** Daša Kokole, Eva Jané-Llopis, Guillermina Natera Rey, Natalia Bautista Aguilar, Perla Sonia Medina Aguilar, Juliana Mejía-Trujillo, Katherine Mora, Natalia Restrepo, Ines Bustamante, Marina Piazza, Amy O’Donnell, Adriana Solovei, Liesbeth Mercken, Christiane Sybille Schmidt, Hugo Lopez-Pelayo, Silvia Matrai, Fleur Braddick, Antoni Gual, Jürgen Rehm, Peter Anderson, Hein de Vries

**Affiliations:** 1 Department of Health Promotion, CAPHRI Care and Public Health Research Institute, 5211 Maastricht University, POB 616, 6200 MD, Maastricht, The Netherlands; 2 Univ. Ramon Llull, 69544 ESADE, Avenida de Pedralbes, 60, 62, 08034 Barcelona, Spain; 3 585567 Institute for Mental Health Policy Research, CAMH, 33 Russell Street, Toronto, ON M5S 2S1, Canada; 4 42584 Instituto Nacional de Psiquiatría Ramón de la Fuente Muñiz, Calz México-Xochimilco 101, Huipulco, 14370 Ciudad de México, CDMX, Mexico; 5 Corporación Nuevos Rumbos, Calle 108 A # 4-15, Bogotá, Colombia; 6 School of Public Health and Administration, 33216 Universidad Peruana Cayetano Heredia, Ave. Honorio Delgado 430, Urb. Ingeniería, S.M.P. Lima - Perú; 7 Population Health Sciences Institute, 5994 Newcastle University, Baddiley-Clark Building, Richardson Road, Newcastle upon Tyne NE2 4AX, UK; 8 Department of Health Psychology, 10198 Open University, Valkenburgerweg 177, 6419 AT Heerlen, the Netherlands; 9 Centre for Interdisciplinary Addiction Research (ZIS), Department of Psychiatry and Psychotherapy, 37734 University Medical Centre Hamburg-Eppendorf, Hamburg, Germany; 10 Addictions Unit, Psychiatry Dept, 16493 Hospital Clínic, Villarroel 170, 08036 Barcelona, Spain; 11 Red de Trastornos Adictivos, 38176 Instituto Carlos III, Sinesio Delgado, 4, 28029 – Madrid, Spain; 12 146245 Institut d’Investigacions Biomèdiques August Pi Sunyer (IDIBAPS), Rosselló, 149-153, 08036 Barcelona, Spain; 13 Institute for Clinical Psychology and Psychotherapy, 9169 TU Dresden, Chemnitzer Str. 46, 01187 Dresden, Germany; 14 274071 Dalla Lana School of Public Health, 149914 University of Toronto, 155 College Street, 6th Floor, Toronto, ON M5T 3M7, Canada; 15 Department of Psychiatry, 149914 University of Toronto, 250 College Street, 8th Floor, Toronto, ON M5T 1R8, Canada; 16 Department of International Health Projects, Institute for Leadership and Health Management, I.M. Sechenov First Moscow State Medical University, Trubetskaya str., 8, b. 2, 119992, Moscow, Russian Federation

**Keywords:** implementation, process evaluation, training, alcohol, depression, screening, primary health care, middle-income

## Abstract

**Background:**

Initial results from the SCALA study demonstrated that training primary health care providers is an effective implementation strategy to increase alcohol screening in Colombia, Mexico and Peru, but did not show evidence of superior performance for the standard compared to the shorter training arm. This paper elaborates on those outcomes by examining the relationship of training-related process evaluation indicators with the alcohol screening practice.

**Methods:**

A mix of convergent and exploratory mixed-methods design was employed. Data sources included training documentation, post-training questionnaires, observation forms, self-report forms and interviews. Available quantitative data were compared on outcome measure – providers’ alcohol screening.

**Results:**

Training reach was high: three hundred fifty-two providers (72.3% of all eligible) participated in one or more training or booster sessions. Country differences in session length reflected adaptation to previous topic knowledge and experience of the providers. Overall, 49% of attendees conducted alcohol screening in practice. A higher dose received was positively associated with screening, but there was no difference between standard and short training arms. Although the training sessions were well received by participants, satisfaction with training and perceived utility for practice were not associated with screening. Profession, but not age or gender, was associated with screening: in Colombia and Mexico, doctors and psychologists were more likely to screen (although the latter represented only a small proportion of the sample) and in Peru, only psychologists.

**Conclusions:**

The SCALA training programme was well received by the participants and led to half of the participating providers conducting alcohol screening in their primary health care practice. The dose received and the professional role were the key factors associated with conducting the alcohol screening in practice.

**Plain Language Summary:** Primary health care providers can play an important role in detecting heavy drinkers among their consulting patients, and training can be an effective implementation strategy to increase alcohol screening and detection. Existing training literature predominantly focuses on evaluating trainings in high-income countries, or evaluating their effectiveness rather than implementation. As part of SCALA (Scale-up of Prevention and Management of Alcohol Use Disorders in Latin America) study, we evaluated training as implementation strategy to increase alcohol screening in primary health care in a middle-income context. Overall, 72.3% of eligible providers attended the training and 49% of training attendees conducted alcohol screening in practice after attending the training. Our process evaluation suggests that simple intervention with sufficient time to practice, adapted to limited provider availability, is optimal to balance training feasibility and effectiveness; that booster sessions are especially important in context with lower organizational or structural support; and that ongoing training refinement during the implementation period is necessary.

## Introduction

Primary health care (PHC) providers are a key group that can encourage adults to adopt a healthier lifestyle, as they have regular access to large portions of the population through routine consultations. One of the key components of a healthy lifestyle is reduction of alcohol use, alcohol being the ninth-largest risk factor for morbidity globally, and fourth in Latin America (GBD 2019 Risk Factors Collaborators, 2020). Screening for patients’ risky alcohol use during a routine check-up in PHC and providing them with advice on cutting down if necessary has a large body of evidence supporting its effectiveness in the reduction of alcohol use (Kaner et al., 2018; O’Donnell, Anderson, et al., 2014). Despite its effectiveness and simplicity, it is often sub-optimally implemented in practice (O’Donnell, Wallace, et al., 2014) and a large body of research deals with how to improve the implementation to achieve better public health outcomes (Anderson et al., 2016; Keurhorst et al., 2016; Heather, 2006).

An implementation strategy consistently shown to be effective in improving the implementation of healthcare interventions in general (Rowe et al., 2018), and alcohol screening in particular (Anderson et al., 2014) is training of the PHC providers. Conversely, lack of appropriate training has emerged as a major barrier to practice; in a recent systematic review of factors influencing PHC providers’ implementation of alcohol screening and brief intervention in primary care practices, lack of training was the most commonly emerging theme among the cited barriers, closely followed by the alcohol-related knowledge and the belief in one's own ability to deliver the intervention - both of which can be also targeted via training (Rosário et al., 2021). However, the majority of the existing alcohol screening research (both in general and training-specific) comes from high-income countries (Rosário et al., 2021). While alcohol screening programmes have been implemented and evaluated in Latin America (Ronzani et al., 2019; Shannon et al., 2021), evaluation of providers’ training research remains scarce (e.g. (Furtado et al., 2008). Training evaluation in other healthcare-related fields focuses on effectiveness rather than the implementation (Smith et al., 2020; Spagnolo et al., 2020; Stokholm Bækgaard et al., 2021; Stoltenberg et al., 2020; Suriyawongpaisal et al., 2020). This paper addresses this gap in the literature and presents the findings of an in-depth process evaluation of using a training package as an implementation strategy to increase alcohol screening by the PHC providers in a middle-income context.

**Figure 1.**
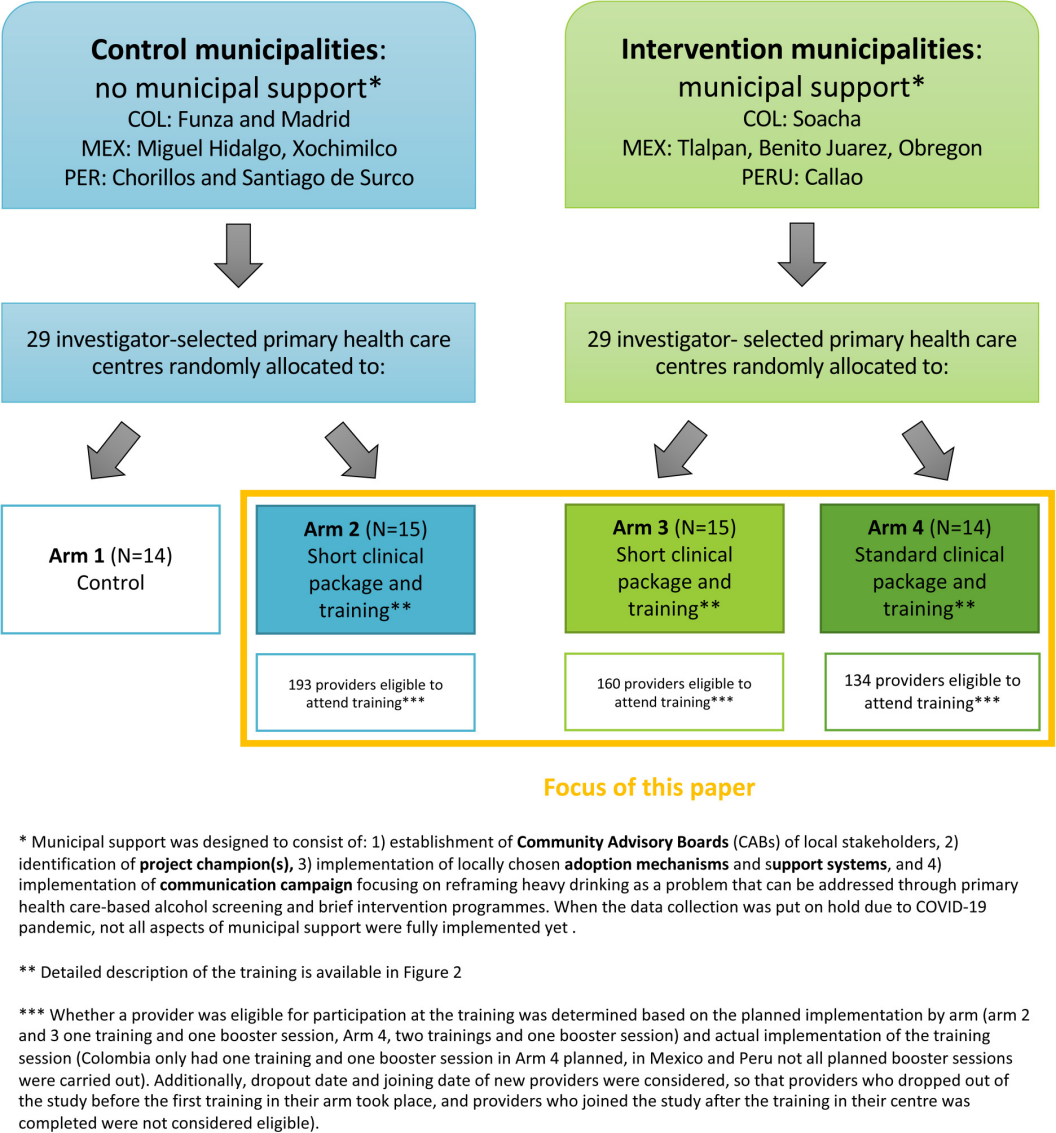
SCALA study design.

SCALA (Scale-up of Prevention and Management of Alcohol Use Disorders in Latin America) is a quasi-experimental study conducted in three middle-income Latin American countries (Colombia, Mexico and Peru), testing whether training PHC providers and providing municipal support (a range of adoption mechanisms and support systems) could support improved implementation of PHC-based screening, advice and treatment for heavy drinking and comorbid depression (Jane-LLopis et al., 2020). In addition to screening for risky alcohol consumption of all patients, and providing advice on cutting down if necessary, SCALA clinical care pathway requires providers to check for depressive symptoms in the heavy drinking patients, as heavy drinking is often comorbid with depression (Bellos et al., 2016), and associated with worsening of depression, including increased suicide risk and impaired social functioning (Boden & Fergusson, 2011). A summary of the study design and the included intervention components by arm is presented in Figure 1, and further detailed description is available in the protocol paper (Jane-LLopis et al., 2020). The results of the outcome evaluation at the primary health care center (PHCC) level have been published elsewhere and the findings suggest that the providers’ training significantly increased the proportion of alcohol screening in adult patients (although the standard training and clinical package was not superior to shorter version) (Anderson et al., 2021), as well as the depression screening rates (O’Donnell et al., 2021). At the time of evaluation, municipal support (as described in Figure 1), was not found to have a significant impact (Anderson et al., 2021), which was likely due to its incomplete implementation, as the implementation had to be put on hold at the 5-month mark due to COVID-19 restrictions.

In this paper, we present the findings from the process evaluation of SCALA training as the implementation strategy already demonstrated to be effective to increase alcohol screening (Anderson et al., 2021). We used the UK Medical Research Council (MRC)'s process evaluation guidance (Moore et al., 2014, 2015) to develop the process evaluation protocol (Jane-LLopis et al., 2020) and guide the aims, focusing on the key issues regarding implementation, mechanisms of impact, and context along with their relationship to the outcome. The main research questions addressed in this paper include:

- What was implemented in terms of training dose, reach, adaptation and fidelity?
- How did the participants respond to the training, and were there any unintended consequences of the training implementation?
- How were the implementation factors, participant response and provider demographics associated with the main study outcome (alcohol screening in practice)?

## Methods

### Design and Setting of the Study

We used the StaRI checklist (Pinnock et al., 2017) to report on the study. The presented data have been collected as part of the broader process evaluation (Jane-LLopis et al., 2020) to support an in-depth understanding of study's primary results (Anderson et al., 2021). We employed mixture of the convergent and exploratory mixed-methods design (Creswell & Plano Clark, 2011), whereby the qualitative and quantitative data were collected in the same period, analyzed separately, but then complemented with additional qualitative data and interpreted together. Some relevant characteristics of the implementation setting are presented in Table 1.

**Table 1.** Description of the setting characteristics in Colombia, Mexico and Peru.

	Colombia	Mexico	Peru
***Setting description***			
*Main country demographics*	• Population: 48 258 494 (2018 data) • 51.2% female • 75.5% living in urban areas • Age distribution: 24.0% under 15, 67% 15–64, 8.8% 65 + ^a^	• Population 119 938 473 (2015 data) • 51.4% female • 76.8% living in urban areas • Age distribution (2010 data): 29.3% under 15, 64.4% 15–64, 6.3% 65 + ^b^	• Population 31 237 385 (2017 data) • 50.5% female • 81.9% living in urban areas • Age distribution: 26.5% under 15, 65.3% 15–64, 8.2% 65 + ^c^
*GDP per capita (2019)^d^*	6508.1 USD	10118.2 USD	7046.8 USD
*Income level (World bank)^e^*	Upper-middle income	Upper-middle income	Upper-middle income
*Participating municipalities*	Intervention (Arm 3 and 4): Soacha (population: 93.154; located in the metropolitan area of Bogota, part of the department of Cundinamarca).^a^ Control (Arm 1 and 2): Funza (pop: 112.254), Madrid (93.154); both located in Western Savanna Province and part of the department of Cundinamarca, 25 km outside Bogota.^a^	Intervention: Tlalpan (650.567)*, Benito Juárez (385.439), Álvaro Obregón (727.034); all one of 16 municipalities of Mexico City. ^b^ Control: Miguel Hidalgo (372.889), Xochimilco (415.007), both one of 16 municipalities of Mexico City.^b^ *two of PHCUs from this municipality are in the control arm	Intervention: Callao (pop: 451.260): Provincial capital and one of the seven districts in Callao province, part of Callao region. Located in the West area of Lima, and borders the Pacific Ocean. ^c^ Control: Chorillos (314.241) and Santiago de Surco (329.152); both one of the 43 districts of Lima province, located in the Lima region, bordering each other. ^c^
*Existing alcohol SBI practice and guidelines*	The alcohol SBI recommendations are included as part of clinical practice guidelines that focus on detection and treatment of alcohol abuse and dependence on primary, secondary and tertiary care level^f^ but there are no official standards. Some of the providers are familiar with the screening instruments.	Official standards establish the obligatory procedures and criteria for mandatory prevention, treatment and control of addictions, which include asking questions on alcohol use^g^ and including this information in the patient's history^h^ specifically in primary health care context.	No explicit guidelines, recommendation for providers to include alcohol screening is implicitly included in general recommendation to perform mental health related screening (alcohol use disorder being considered as one of subcategories)^i^
*Organisational context in the participating PHCCs ^j^*	In the intervention arm, the leadership was very supportive of the project, and there was leadership directive for providers’ participation and assigned time to attend the trainings.	The organisational context depended on the centres, with varying levels of leadership support. In all of the participating centres there was existing screening practice due to standards described above, and providers were familiar with the screening instruments and often also have experience with its application.	There was no consistent leadership directive in the centres. None of the participating centres had an existing screening practice and providers were generally not familiar with screening instruments.
*Provider recruitment details*	In all arms, the providers were chosen for participation by their superiors.	The recruitment varied by centre, with some providers being selected for participation and some providers volunteering to participate.	The providers had to volunteer to join the project.

^a^ DANE (2018). Censo nacional de población y vivienda. Proyecciones de población. Available from: https://www.dane.gov.co/index.php/estadisticas-por-tema/demografia-y-poblacion/proyecciones-de-poblacion [accessed 23.9.2020]. ^b^ INEGI (n.d.). Banco de indicadores, 2015. Available from https://www.inegi.org.mx/app/indicadores/?t = 0070&ag = 09014##D00700060 [accessed 23.9.2020] ^c^ INEI (2017). Censos nacionales 2017: XII Censo de Población, VII de Vivienda y III de Comunidades Indígenas. Sistema de Consulta de Base de Datos. Available from: http://censos2017.inei.gob.pe/redatam/ [accessed 23.9.2020] (data from 2017). ^d^ IMF (2019). World Economic Outlook: https://www.imf.org/en/Publications/SPROLLS/world-economic-outlook-databases. ^e^ World bank (n.d). World Bank Country and lending groups: https://datahelpdesk.worldbank.org/knowledgebase/articles/906519. ^f^ Ministerio de Salud y Protección Social. Guía de práctica clínica para la detección temprana, diagnóstico y tratamiento de la fase aguda de intoxicación de pacientes con abuso o dependencia del alcohol - 2013 Guía No. 23 [Internet]. 2013. Available from: https://www.minsalud.gov.co/sites/rid/Lists/BibliotecaDigital/RIDE/INEC/IETS/GPC_Completa_OH.pdf. ^g^ Norma Oficial Mexicana NOM-028-SSA2-2009 para la prevención, tratamiento y control de las adicciones [Internet]. 2009. Available from: http://www.conadic.salud.gob.mx/pdfs/norma_oficial_nom.pdf. ^h^ Norma Oficial Mexicana NOM-004-SSA3-2012 del expediente clínico [Internet]. 2012. Available from: https://www.cndh.org.mx/DocTR/2016/JUR/A70/01/JUR-20170331-NOR26.pdf. ^i^ Ministerio de Salud Perú. Plan nacional de fortalecimiento de servicios de salud mental comunitaria 2018-2021 [Internet]. 2018. Available from: http://bvs.minsa.gob.pe/local/MINSA/4422.pdf. ^j^Information provided by local research partners based on field visits.

### SCALA Training Curriculum

**Figure 2.**
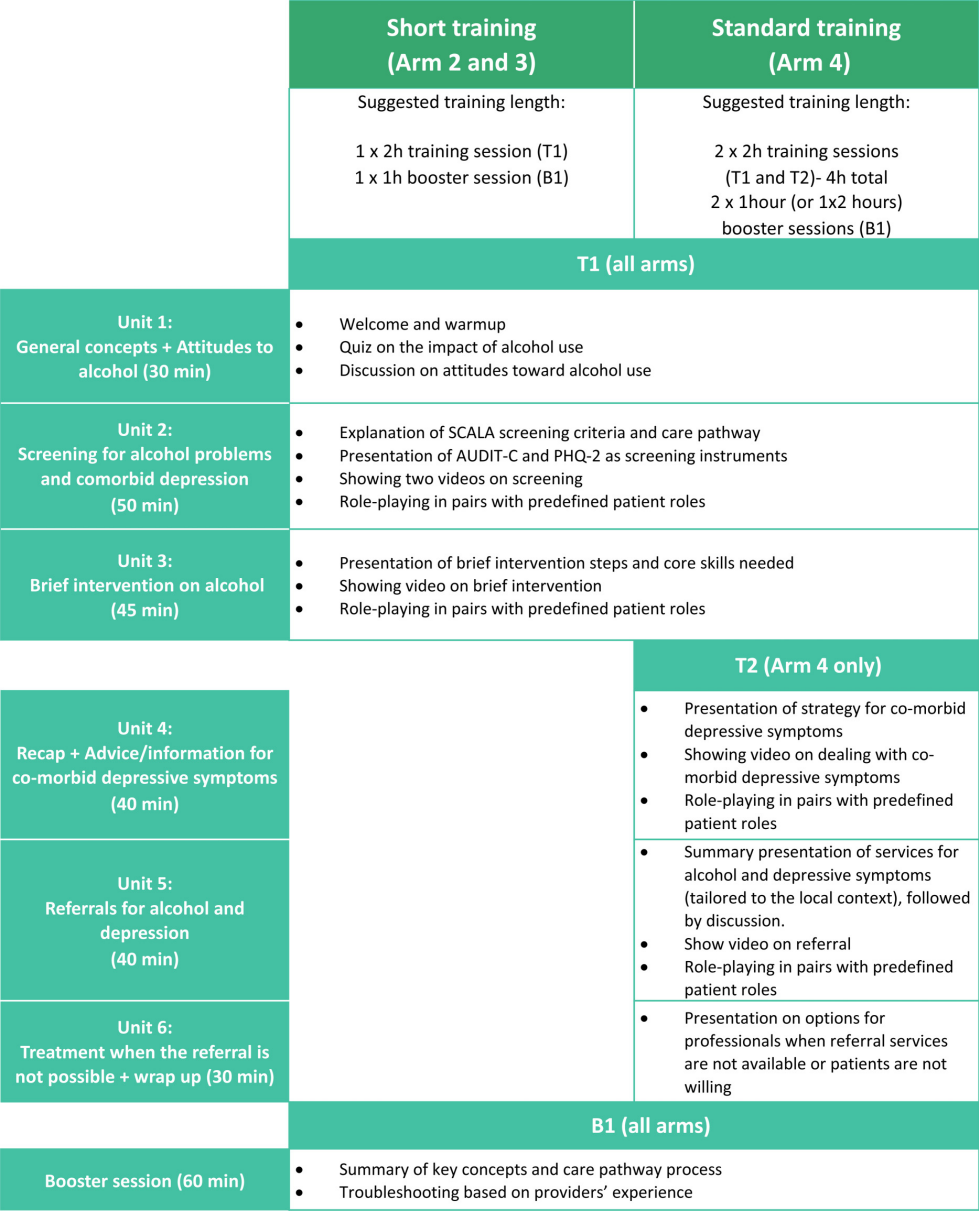
SCALA training curriculum.

Figure 2 describes the SCALA training curriculum. Two versions of the training package were developed, a short and standard version, to be tested in different arms. Both were designed to be flexible and easy to adapt to the country and local context. The training package consisted of four products: the training manual; the training course presentations; the training videos, and the Train New Trainers sessions. The key differences between the short and standard training package were different care pathways (short vs. standard, as described in the study protocol (Jane-LLopis et al., 2020)), and a different set of videos. Both training sessions focused on alcohol screening and advice, and the standard training additionally emphasized dealing with co-morbid depressive symptoms, and alcohol referral and treatment options. The core of the training sessions was based on two main components: videos showing PHC providers delivering the protocol in practice and role-plays using the developed materials. The theoretical background for this approach comes from social cognitive theory (Bandura, 1977), where both vicarious learning (through modelling) and enactive mastery (practicing the skills yourself) are key components of increasing self-efficacy. In practice, this approach has been used in previous similar projects (e.g. ODHIN, PHEPA) with demonstrated effectiveness (Anderson et al., 2016).

Before training implementation, Train New Trainers course took place in Bogota, Colombia in May 2018, attended by the local professionals (doctors, psychologists, addiction specialists) from the three countries (future trainers, N  =  16). The training was conducted by an addiction specialist with several years of experience in implementing brief interventions and training delivery. A detailed description of the full training package and the process of its development is available in Supplementary file 1, along with the links to all the training materials (SCALA, 2021).

### Participants

PHC providers of any professional role from the participating centers were eligible for participation in the SCALA study upon signing informed consent. Information on provider recruitment is described in Table 1. In this paper, we included the providers from 44 PHCCs in Arms 2, 3 and 4 who were eligible to be trained either in the baseline period, or through booster sessions taking place in the first five months of the implementation period of the study. Some centers were in their 6^th^ or 7^th^ month of implementation when data collection was halted due to the start of the COVID-19 pandemic and were still recruiting new providers at that point, but we used the 5-month mark to allow for better alignment with the data presented in the main outcome paper (Anderson et al., 2021). Providers from the 14 PHCCs in Arm 1 (control group) who did not receive training, as well as providers who attended the training without signing the informed consent to participate in the study, were not included in the data collection and analysis.

### Measurements

The selection of constructs to inform the training process evaluation was based on the UK MRC process evaluation guidance (Moore et al., 2015). Table 2 presents an overview of the key measured constructs, the data sources used for their assessment, and information on data integration. We included also an outcome indicator – using SCALA clinical package in practice at least once. As the SCALA care pathway was designed for the providers to screen for alcohol consumption first and only use depression screening if the patient was identified as drinking at a risky level, alcohol screening was used as a proxy measure to indicate the use of the SCALA clinical package.

**Table 2.** Key measured constructs for the process evaluation of the training.

Construct measured	Definition (MRC)^a^	Operationalization	Data source used	Data integration
*Implementation*				* *
Dose	Quantity of what has been delivered in practice (how much intervention is delivered)	Actual amount and length of the training per country and arm (dose delivered) Number of hours and sessions provider participated in the training, per country and arm (dose received)	Training documentation, attendance lists	Quantitative data only, information on dose delivered combined with individual attendance information to calculate dose received
Reach	The extent to which the target audience comes into contact with the intervention	Number and % of all providers recruited for the study and eligible for the training that participated in the trainings^b^ calculated for each training session separately and overall, per country and per arm, Representativeness of the reached population assessed through a comparison of demographic characteristics (age, gender, professional role) between the reached population and non-reached population.	Training documentation, consent form information	Quantitative data on reach complemented with qualitative on reasons for provider non-participation (as obtained through interviews)
Adaptation	Alterations made to intervention to better fit the context	Description of parts of the training that were adapted	Observation forms, trainer self-report forms	Results triangulated from both data sources and complemented with qualitative data on reasons for changes
Fidelity	Quality of what is delivered or consistency of what is implemented with the planned interventions	Delivery of training's active ingredients (videos and role-plays): complete, partial or none.	Observation forms, trainer self-report forms	Results triangulated from both data sources and complemented with qualitative data on reasons for changes
*Mechanisms of impact*				* *
Participant response	How do participants interact with the intervention	Satisfaction with the training Perceived utility of the training Suggestions for improvement	Post-training questionnaire, interviews	Quantitative data complemented with qualitative data on provider's impressions and suggestions for improvements and qualitative data from trainer interviews
Unintended consequences	Unanticipated pathways and consequences	Emerging side-effects of delivering trainings in the PHCC	Interviews	Qualitative data only
*Context*				* *
Demographics	Factors external to the intervention which impede or strengthen its implementation or effects	Country, age, gender, professional role of providers	Consent forms	Quantitative data only
*Outcome*				* *
Outcome behaviour		Participating providers doing alcohol screening in practice at least once	Tally sheets	Quantitative data only, integrated with dose, participant response and demographic information

^a^ In this paper, term intervention (as used in the MRC definition) refers to the training package. **^b^** Whether a provider was eligible for participation at the training was determined based on the planned implementation by arm (arm 2 and 3 one training and one booster session, Arm 4, two trainings and one booster session) and actual implementation of the training session (Colombia only had one training and one booster session in Arm 4 planned, in Mexico and Peru not all planned booster sessions were carried out). Additionally, dropout date and joining date of new providers were considered, so that providers who dropped out of the study before the first training in their arm took place, and providers who joined the study after the training in their centre was completed were not considered eligible). Satisfaction with the training. Perceived utility of the training. Suggestions for improvement.

### Data Sources and Collection

Details on the data sources are presented below along with data collection procedure description.

#### Training Documentation

Training logbooks were completed by the local research partners throughout the implementation period and contained information on the delivered training (date, time and training location) and participating providers (based on the information from the signed attendance lists). This allowed us to assess the dose and reach of the training; the latter also in combination with demographic data gathered during study recruitment.

#### Post-training Questionnaire

The questionnaire assessed participant response to the training. Participants answered a set of questions on a 5-point Likert scale (1-Very negative to 5-very positive for satisfaction and 1-Not very useful to 5-Very useful for perceived utility) and additionally had space to provide open-ended answers. Providers completed the pen-and-paper questionnaires at the end of the training session in the period between August 2019 and March 2020. Questionnaires contained the predetermined provider ID to guarantee anonymity and individual traceability.

#### Observations

The training sessions were observed by previously trained local research team members. In Colombia and Peru, all the sessions were observed. In Mexico, one session per arm was observed due to resource limitations. Researchers used a structured observation form containing both quantitative indicators (e.g. checklists to mark the delivery of listed active ingredients to assess fidelity), and there was room for qualitative observations (e.g. providing an additional explanation in case of non- or partial execution of activity).

#### Self-report Forms

The trainers completed the self-report form after each delivered training, providing information on which components were delivered and whether they adapted the training, along with explanations. Fidelity and adaptation questions in the observation and self-reports form were the same to allow for data triangulation.

#### Interviews

The interview topic guide was tailored to the country to complement the information obtained from other sources based on previous familiarization with data from other sources by the interviewer. The interviews were conducted after data from other sources (both qualitative and qualitative) was already partially analyzed. The initial interview guides were developed as part of process evaluation protocol development, and were adapted to reflect any additional issues that emerged during the data familiarization and preliminary analysis phase. In total, three group interviews (one per country) were conducted with a total of nine participants (two in Colombia, two in Peru and five in Mexico). All participants were either trainers (N  =  7) or training organizers (N  =  2) in their countries.

#### Tally Sheets (As Outcome Data)

During the implementation period, providers completed a tally sheet each time they conducted a screening. Those were collected from the PHCCs on monthly basis by the local researchers.

The majority of the data was collected between August 2019 and March 2020, with exception of the interviews, conducted between November and December 2020. The first part of the data was collected by the local research teams (one in each of the countries), and all the evaluation materials were transferred electronically to the evaluation coordinator using 256-bit ‘Advanced Encryption Standard’ (AES). The online interviews were conducted by the process evaluation leader, audio-recorded with the consent of all participants, transcribed in Spanish and translated to English. All recordings were destroyed after transcription.

### Data Analysis

Quantitative data were analyzed with SPSS 25 (IBM Corp., 2017). Frequencies and descriptive statistics (mean, standard deviation) were calculated and group comparisons were made using Mann-Whitney U and Chi-square tests - overall, and by country or arm where applicable. Qualitative data was analyzed based on a combination of inductive and deductive coding using Atlas.ti 8.4 (Scientific Software Development GmbH, 2019). The main framework for deductive coding of qualitative parts of post-training questionnaires, observation forms and self-report forms were the categories based on the MRC guide as presented in Table 3, and within those categories, themes were coded inductively. Any discrepancies between information in observation and self-report forms were resolved through discussion with the local research teams. To code the interviews, independent double coding was conducted based on the framework by two researchers (DK and AS), PhD candidates with a background in health promotion/health communication and implementation science, followed by a coding comparison and summary of the main emerging themes. The lead author integrated the quantitative and qualitative data by the framework category with the purpose of complementarity (elaboration and clarification of the results obtained through one method with results from another) (Greene et al., 1989). Table 2 gives a more detailed indication on how the data were integrated at the point of analysis.

**Table 3.** Reach and dose by country.

	Colombia	Mexico	Peru
*Reach*	67 (89.3% eligible) providers attended at least one session	139 (65.0% eligible) providers attended at least one session	146 (73.3% eligible) providers attending at least one session
*Dose delivered*	Total: 16 sessions (7 T1 + 9 B)	Total: 26 sessions (18 T1,T2 + 8 B)	Total: 33 sessions (20 T1,T2 + 13 B)
	3.5 h (1.5 T1 + 2 B) - Arm 2 and 3	3 h (2 T1 + 1 B) - Arm 2 and 3	4 h (2 T1 + 2 B) - Arm 2 and 3
	4 h (2 T1 + 2 B) - Arm 4	5 h (2 T1 + 1 T2 + 2 B) - Arm 4	6 h (2 T1 + 2 T2 + 2 B) - Arm 4
*Dose delivered - COVID-19 impact*	All training and booster sessions delivered before the start of the COVID-19 pandemic.	Six booster sessions not delivered in part due to the COVID-19 pandemic (sessions were hard to schedule because of lower priority in the centres, and then had to be further postponed to COVID-19)	Two booster sessions not delivered in part due to the COVID-19 pandemic (sessions were first postponed due to scheduling issues and lack of trainers’ time and then had to be cancelled due to COVID).
*Dose received*	On average, the providers participated:	On average, the providers participated:	On average, the providers participated:
	2.3h Arm 2	1.9h Arm 2,	2.6h Arm 2,
	2.4h Arm 3	2.1h Arm 3,	2.6h Arm 3,
	3.1h Arm 4	2.8h Arm 4	4.2h Arm 4

*Note*. T1 – first training session (all arms), T2 – second training session (only Arm 4), B – booster session.

### Ethics

The study has been reviewed and approved by the research ethics board at the TU Dresden, Germany (registration number: ‘EK 90032018’), and by the ethics boards in Colombia, Mexico, and Peru. All participating providers signed informed consent upon study recruitment.

## Results

**Figure 3.**
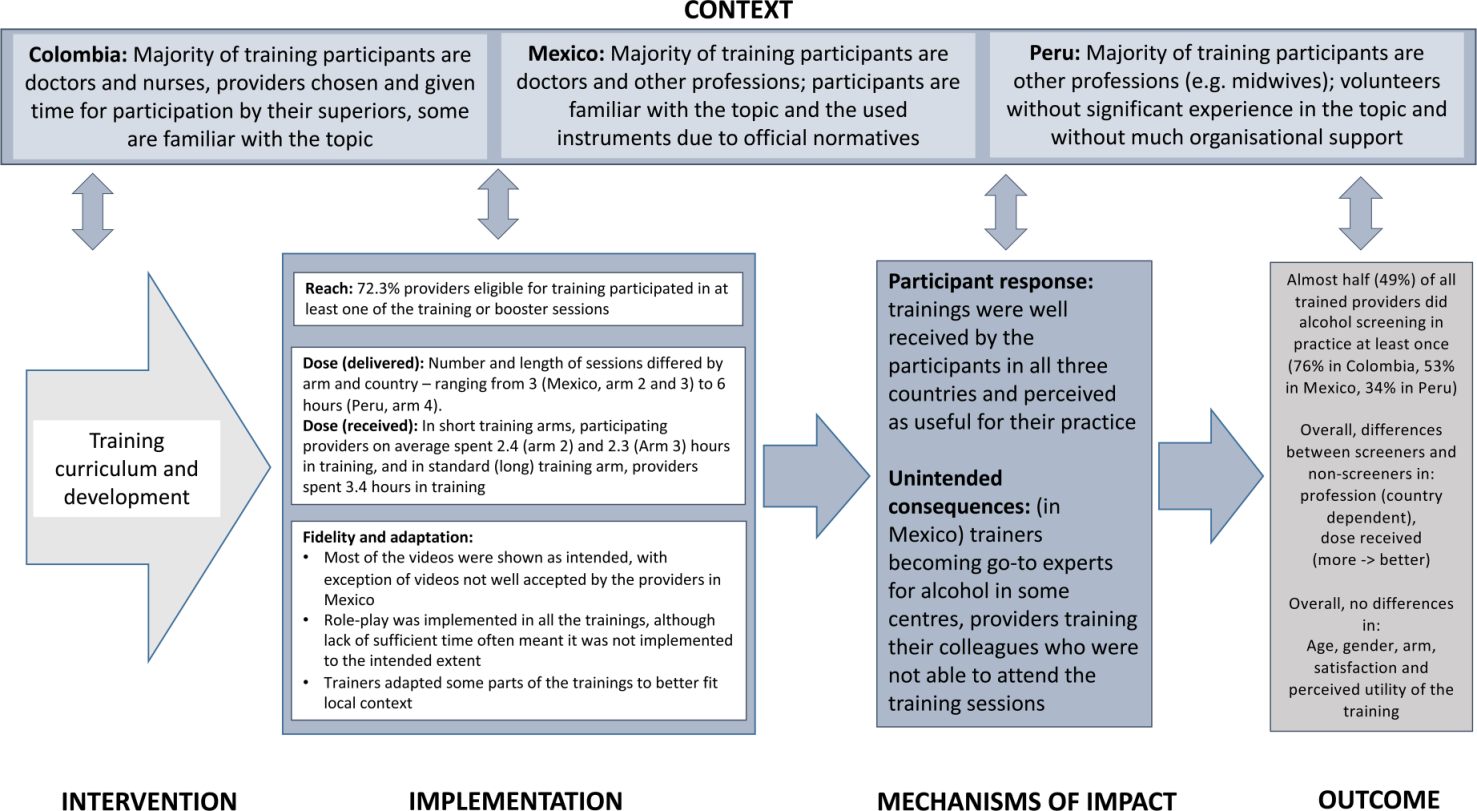
Summary of key findings of SCALA training process evaluation.

The results of the key process indicators and their relationship with the outcome are presented below. Due to the large amount of collected and analyzed data, only the key tables are included in the results section, remaining tables are available as supplementary material (Supplementary file 2). A summary of the key findings is presented in Figure 3.

The training sessions took place between August and November 2019, and booster sessions took place between January and March 2020. In total, 45 training sessions and 30 booster sessions were delivered by twelve trainers (three from Colombia, five from Mexico, and four from Peru). Most of the activities were carried out before the restrictions due to the COVID-19 pandemic (Table 3).

### Reach and Dose

First, we calculated reach (how many providers attended the training), dose delivered (how much training was offered) and dose received (how much training the providers attended) based on the information from the training documentation and attendance lists. A summary of the main reach and dose indicators is presented in Table 3, with complete information on reach and dose by country and arm available in Supplementary file 2, Table S2. Overall, almost three quarters (72.3%) of all eligible providers attended at least one of the delivered training sessions, with the highest percentage in Arm 4 (76.9%), followed by Arm 2 (74.1%) and Arm 3 (66.3%). In Arm 2 and 3, providers in all three countries had the opportunity to attend two sessions (one main and one booster), and in Arm 4, three sessions (with exception of Colombia, where two sessions were provided). Length of sessions differed by arm and country, ranging from three (Mexico, arms 2 and 3) to six hours (Peru, arm 4). The average number of hours attended across countries was highest in standard training in Arm 4 (3.4), and was comparable between short training arms (Arm 2 and 3); 2.4 and 2.3 h respectively. On average across arms, providers in Colombia spent 2.7 h in training, in Mexico 2.2 h and in Peru, 3.1 h.

Comparing the attending and non-attending providers on demographic characteristics (Supplementary file 2, Table S3) showed no overall difference by age or professional role, but a higher percentage of eligible women attending compared to eligible men (74.8% vs. 65.1%, χ^2^  =  4.40, p  =  0.036). In Mexico, we found significant difference regarding professional role (χ^2^  =  8.24, p  =  0.041), as all the eligible psychologists, but only over half of the nurses attended the training.

As part of the interviews, we asked the local training teams about what they perceived as reasons for some providers not attending the training. Respondents indicated that lack of attendance did not always mean a lack of interest in the project; alternative reasons for non-attendance included conflicting work obligations, or not being present at work on that day (e.g. some providers only worked weekends). Additionally, in Peru, there was a three-month gap between recruitment and training, so a possible reason suggested by the trainers was that some providers forgot they registered. All providers who were allocated to training arms in SCALA were sent training presentation slides and materials however, and in Mexico, some received additional guidance from colleagues who attended the training (see also Unintended consequences).

### Fidelity and Adaptation

Table 4 presents the extent to which the main elements of the training were delivered, including the *explanation of basic concepts, videos* and *role-plays,* based on the data obtained through observation and self-report forms. Overall, the *explanation of the basic concepts* was delivered fully. *Videos* were to large extent shown as intended in Colombia and Peru, except in a few cases where trainers ran out of time. In Mexico, after showing videos at the initial 8 training sessions, comments from providers suggested that the videos did not sufficiently reflect the organizational context, therefore the training team decided to replace the videos with hypothetical cases suggested by the providers themselves.

**Table 4.** Overview of training fidelity and adaptations by country.

	Colombia	Mexico	Peru
*Fidelity - short training*			
Explanation of basic concepts: Explanation screening criteria Present AUDIT-C and PHQ-2 Present steps of alcohol brief intervention Introduction of core skills	Completed fully	Completed fully	Completed fully
Showing videos screening video (alcohol screening – negative) screening video (alcohol screening – positive; depression screening – negative) brief intervention video (brief intervention for alcohol)	Completed fully	Completed partially – based on feedback from first few 8 trainings, screening videos were not shown in remaining training sessions (feedback from the participants was is that it did not reflect the Mexican context)	Completed fully – with exception of one case where brief intervention video was not shown due to lack of time
Performing role plays Screening role play in pairs, with exchange of roles Brief intervention role play in pairs, with exchange of roles	Completed partially – due to lack of time both role-plays had to be merged in most training sessions	Completed partially – both role plays were done, but no exchange of roles	Completed partially – in some trainings, role plays had to be merged due to lack of time
*Fidelity - standard training*			
Explanation of basic concepts Session 1: Explanation of screening criteria Presentation AUDIT/AUDIT-C and PHQ-2/9 Presentation steps of alcohol brief intervention Introduction of core skills Session 2 Explanation of strategy for the management of co-morbid depressive symptoms Presentation of the summary of services for the treatment of depressive symptoms and problematic alcohol use. Presentation of treatment and follow-up options when referral is not possible	Completed fully	Completed fully Note: In Mexican centres the referral pathways are well defined, so the trainer did not have to spend much time on it	Completed fully
Showing videos Session 1: alcohol screening – negative alcohol screening – positive; depression screening – negative screening and brief intervention – alcohol and depression positive brief intervention for alcohol Session 2: brief intervention for alcohol and depression referral for alcohol problems and co-morbid depressive symptoms patient not accepting referral	Session 1: Completed fully Session 2: Completed fully – exception one training where one video could not be shown due to lack of time	Session 1: Completed fully Session 2: Completed partially: based on feedback from first few 8 trainings, screening videos were not shown in remaining training sessions	Session 1: Completed fully Session 2: Completed fully
Performing role plays Session 1: Screening role play in pairs, with exchange of roles Brief intervention role play in pairs, with exchange of roles Session 2: Brief intervention for alcohol and depression role play in pairs, with exchange of roles Referral role play in pairs, with exchange of roles	Session 1 and 2: completed partially - All role plays were merged, focus was on the first one. Role-plays from session 2 not done in one of the trainings (lack of time). Overall less time dedicated for role-plays	Session 1: completed fully; if no time then postponed to session 2 Session 2: Completed partially: both role plays were done, but no exchange of roles	Session 1: Completed fully Session 2: Completed fully

The *role-plays* were always delivered, but were commonly shortened due to lack of time, meaning that the participating providers only practiced part of the activities as a health professional, and were experiencing the remaining activities in the patient's role. In all three countries, a lack of time for role play was evident in the short training session. Besides the adaptation of role-plays and video demonstration, other components adapted by the trainers to better fit the local context were the slide deck (to reflect the changed sequences or to reduce the amount of information on one slide), as well as the introduction activities (knowledge quiz and discussion on attitudes towards alcohol).

### Participant Response

The participants completed the evaluation questionnaire at the end of the training (response rate: Training 1 (T1) 95%, Training 2 (T2), 83%, Booster 1 (B1) 77%). All the participants highly rated their overall experience with the course for each of the training or booster sessions (above 4 on a 5-point scale). There was no difference between countries in overall experience with the course in either Training 1 or the booster session. In Training 2, providers in Peru had higher ratings of the overall experience with the course compared to providers from Mexico (H(1)  =  7.28, p  =  0.007), although both ratings were high. In Colombia, the providers were on average slightly less satisfied with the location and venue in the T1 than in Mexico and Peru (H_location_(2)  =  15.97, p < 0.001; H_venue_(2)  =  22.87, p < 0.001). No other major differences between the ratings of the course were found. Full post-training questionnaire results by country and arm are available in Supplementary file 2, Tables S4-S7.

In the questionnaire, the providers could also leave open-ended comments about the training, and their answers are summarized in Supplementary file 2, Table S8. Several participants expressed that they found role-plays helpful and would appreciate more practice and examples, including personalized feedback. Overall, wishing to have more time available for training was a commonly occurring comment (except for the 2^nd^ session in Mexico). Some providers also suggested videos could better reflect the local reality. Concerning logistics, some Colombian providers noted that training location training should be closer to their workplace and the training venue could be more comfortable. Some Peruvian providers wished for more sign-up time slots, as they are working on different schedules, and training reminders. Another theme among Peruvian providers was the importance of social support– both appreciating meeting other providers with similar interests, and wishing more providers would join the training. The interviewed trainers also corroborated the providers’ reports on the importance of the opportunity to practice the skills through role-plays, and emphasized the impact of familiarity with the used instruments – less familiarity was indirectly associated with less time to practice due to more time necessary for explanation.

### Unintended Consequences

No negative unintended consequences were detected in any of the countries, but two positive unintended consequences emerged in Mexico. Interviews suggested that the participating trainers established a good relationship with some PHCCs through their work with SCALA, which led to continued collaboration also beyond the scope of the training and established them as ‘go-to’ local experts on the topic of alcohol (for example, resulting in invitations to speak at relevant events). Additionally, in some Mexican PHCCs, it emerged that the liaising providers (contact persons, who were most closely engaged with SCALA activities) engaged themselves to provide additional explanation and training to their colleagues who were not able to attend the training. Thus, also those providers received information and training from their attending colleagues, broadening the reach of the training.

### Relationship of Process Evaluation Variables with the Outcome

**Figure 4.**
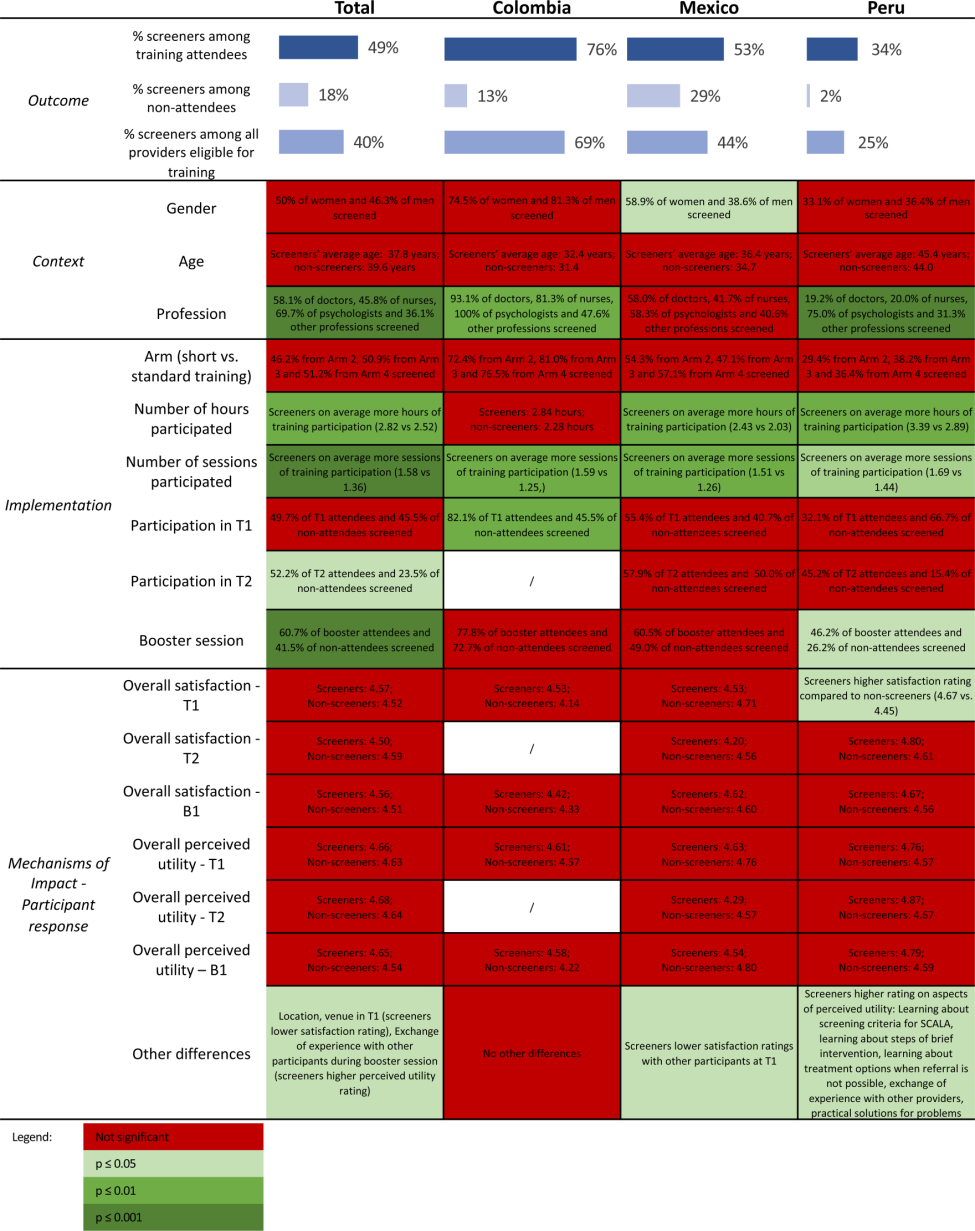
Comparison of screeners and non-screeners on process evaluation variables.

To assess the relationship of context (demographic factors), implementation and mechanisms of impact (participant response) with the outcome, we considered the sample of providers attending minimum one training and participating in minimum one of the five implementation period months (N  =  352). We compared the providers screening in practice at least once (“screeners”, N  =  173) with providers not doing any alcohol screening (“non-screeners”, N  =  179) on characteristics reflecting the broad categories of the MRC framework, using appropriate univariate statistic test (Chi Square or Mann-Whitney U). Results are summarized in Figure 4, with complete analysis available in Tables S9-S11 in Supplementary file 2.

Comparison of the two groups by demographic characteristics showed no significant difference in age (although in all three countries the screeners were on average slightly older than non-screeners). Significant gender differences appeared only in Mexico, where 58.9% of women and 38.6% of men among the training attendees screened (χ^2^  =  4.96, p  =  0.026). There was also a significant difference by profession in Colombia and Peru; in Colombia, both doctors and psychologists were more likely to screen after attending the training (χ^2^  =  14.53, p  =  0.002), although it should be noted that in absolute terms doctors represented the largest part of training attendees. In Peru, the psychologists among the training attendees were more likely to screen than any other profession (χ^2^  =  9.64, p < 0.001).

When comparing the screeners and non-screeners on implementation factors, there was a clear trend in the total sample of screeners spending more time in training, both in terms of hours and sessions participated (2.82 vs. 2.52 h of training participation, p  =  0.003; and 1.58 vs. 1.36 training sessions (p  =  0.001). There was no difference by arm, meaning the providers receiving the standard training were not more likely to screen than providers in the short training arms. Comparison by the participant response showed no difference between screeners and non-screeners based on their satisfaction and perceived utility of the training (except for overall satisfaction with the training in Peru).

Additionally, we also looked at alcohol screening among the non-reached providers (providers eligible for, but not present at any of the trainings). Overall, 18% of providers not present at any of the training sessions still screened in practice, most of them from Mexico.

## Discussion

This paper considered the process indicators related to training primary health care providers in Colombia, Mexico and Peru to deliver alcohol management and depression programme, as well as the relationships of those indicators with the primary outcome behavior, alcohol screening. To our knowledge, this is one of the first papers examining training as an implementation strategy through the process evaluation lens while including a multi-site comparison in the middle-income context.

### Process Indicators Considerations

Reach of the programme was high, with 72.3% of the eligible providers attending at least one of the offered trained sessions. In Mexico, reach was lower compared to the other two countries, but was broadened by participating providers training their non-participating peers, which is reflected also in a relatively high percentage of screeners among providers not attending the training. The country-dependent dose delivered shows that the length of the training was adapted based on the countries’ needs and familiarity of the target population with the topics (e.g. in Mexico, the depression part could be shortened as providers were already familiar with the topic through the World Health Organization's Mental Health Gap Action Programme trainings (World Health Organization, 2008), whereas for most participants in Peru the topic of alcohol screening was completely new (Kokole et al., 2021).

Fidelity of the training was related to dose; e.g. lack of time was mainly reflected in role-plays being shortened; and also to participant response: in Mexico, the videos were less well accepted in the initial trainings and were replaced with practicing with hypothetical cases. Despite assessing fidelity as part of the process evaluation, the perspective of the research and training teams when developing and implementing the trainings was aligned with the dynamic sustainability framework (Chambers et al., 2013), which suggests that quality improvement is more important than quality assurance, and considers that intervention can not be completely optimized prior to implementation,, but can instead be improved through ongoing development, evaluation and refinement in diverse contexts. From this perspective, decisions of local training teams to leave out the videos that were not well received do not represent failure to adhere to the manual, but continued refinement of the training to better fit the local context – “innovation” rather than “drift” in terminology of Bumbarger and Perkins (Bumbarger & Perkins, 2008). The activities remained aligned with theoretical background (Bandura, 1977), as suggested by Moore et al. (Moore et al., 2014), just shifting the focus from vicarious learning to enactive mastery.

### Process Variables Relationship with Outcome Considerations

The developed training was shown to be successful in getting the providers to screen for risky alcohol use (Anderson et al., 2021), thus we also explored which process indicators differed between the screeners and non-screeners. Two of them stood out by relevance: dose received, and professional role.

Overall, screeners received more training (both in terms of length and number of sessions), which points to the clear importance of the dose received (within the country – between countries the amounts of training differed for reasons mentioned earlier). The dose received, however, includes attendance of booster sessions which took place during the implementation period. This means that it's possible that providers who already started screening after the first training were more likely to join the booster sessions, therefore entering a positive feedback loop (Petticrew et al., 2019) – but a more precise time series analysis would be necessary to examine these dynamics and see how that impacted the total number of screenings, which is beyond the scope of this paper. Given the difference between screeners and non-screeners in booster session attendance, booster sessions were important in all three countries, but the importance (inferred from the largest difference) was strongest in Peru, where providers had the least support from other sources and the least familiarity with the topic. In Colombia, there was organizational support, as enrolment in the study took place on the organizational level, and in Mexico, many providers had previous experience and support of health system policies, being expected to include alcohol use in the patient clinical history. This also aligns with the feedback of the Peruvian providers on the importance of social support and appreciation of encountering other providers with similar interests in the training – booster sessions served as additional support in an unsupportive context.

If the dose received seemed to be similarly important across the three countries, this was not the case for the professional role: the country-level dynamics were different, also due to different sample composition. In Colombia, most of the providers were doctors or nurses, with few psychologists, and all of those roles were more likely to screen compared to other professions. In Mexico, both doctors and psychologists were more likely to screen compared to nurses and other professions (although psychologists accounted only for a small proportion of the sample). In Peru, psychologists were more likely to screen than doctors, nurses or other professional roles. These differences perhaps reflect the differences in the country health systems and roles of professionals (specifically for substance use, but also for mental health more broadly) – e.g. in Peru substance use is often framed as part of mental health and alcohol screening is still considered as a domain of psychologists (Cavero et al., 2018), and in Colombia, PHC providers do not always consider being well equipped for dealing with mental health related (“emotional”) topics (Shannon et al., 2021).

For two indicators, no differences were found: arm and participant response (satisfaction and perceived utility). No difference in the arm allocation between the screeners and non-screeners means that extra training session received by Arm 4 did not have an impact on the outcome. While this seems contradictory to the dose result above, a possible explanation is that we only looked at the first step in SCALA protocol use, which is alcohol screening, while the second training session in Arm 4 emphasized the depression part of the care pathway. Another possible explanation for the discrepancy between the two results (arm vs. dose) could also be the greater difficulty of Arm 4 training content, as providers had to master a more complex care pathway, and thus extra time in training did not translate into more practice in alcohol screening. The finding that longer and more complex intervention did not translate into more screening is aligned both with the theory (Rogers’ innovation complexity theorized to lead to lower adoption (Rogers, 2003)), as well as to the evidence from primary care practice (Lau et al., 2016).

Furthermore, satisfaction with training and perceived utility for practice was not related to the outcome; possibly due to the ceiling effect, as all of the ratings were high, also rendering any differences on single items (mostly found in Peru) less practically relevant. However, a recent paper examining screening and brief intervention training effectiveness found that course completion satisfaction was not associated with immediate screening, but with the amount of screening in 12 months (Acquavita et al., 2021). Therefore, further analyses at the end of the project could be useful to associate those process indicators with the total amount of screening conducted.

### Strengths and Limitations of the Study

The main strength of this process evaluation is that it employed a range of methods, combining both quantitative and qualitative insights, which enabled a better understanding of training implementation and outcomes through data integration and corroboration through data triangulation. While we managed to collect a large amount of data in a hard-to-reach setting, there were resource and feasibility restrictions which led to some data collection limitations. For example, the only feedback received from providers was through the post-training questionnaire, which might miss some nuances of their experience. Furthermore, it was not feasible to reach all the non-attending providers, therefore data on reasons for their non-attendance had to be collected through observations from the trainers and training organizers. Finally, the number of interviews was also small and unlike to reach saturation. While not necessarily a limitation of the study per se, the local research and training teams also raised the issue of the interaction between process evaluation and training implementation – in the already scarce time available to deliver the trainings, several evaluation activities also had to be integrated, such as checking the attendance, and completing the post-training questionnaires. Another issue to be noted is the use of mechanisms of impact as a studied category – based on the MRC framework (Moore et al., 2015), we included participant response and unintended consequences as subcategories in this paper, but not variables possibly mediating the outcome (such as knowledge or attitudes), as those will be examined separately. Last, in terms of outcome, we did not make adjustments to the amount of consultations per provider. The reason for this was that we currently only looked at whether the providers did any screening, rather than how much. Further data analysis is needed to unpack the dynamics of the amount of screening throughout the implementation of the whole SCALA project, which was beyond the scope of this paper.

### Implications for Practice

Based on the results of our process evaluation, we collected a number of key learning points which might be relevant for further practice for training implementation in middle-income contexts (Table 5).

**Table 5.** Key recommendations based on learning points from SCALA training process evaluation.

How to increase the rates of alcohol screening through provider training:
At the individual level, dose of training is important – the more of the offered training the provider receives, the better. Increased length of the training is beneficial, unless it comes at the cost of increased complexity of the intervention: a combination of simple intervention with enough time to practice at the training is optimal. On the other hand, dose needs to be balanced with providers’ availability; in our case, more than 2 h of training at the time would not be feasible. At country level, the amount of necessary training depended on the existing knowledge and familiarity with the topic of the providers, therefore the length should be adapted to the country context. Opportunity to practice, for example through role-playing, is considered helpful by the providers; thus, allocating sufficient time for it within the training session is important Booster sessions can serve an important role in encouraging a positive feedback loop in providers’ behaviour, as they are more likely to be attended by the motivated providers who already started with implementation and need additional support. Booster sessions might be especially important in the context where less organizational or structural support is available.

Despite the success of the training, half of the participating providers did not screen in practice, indicating that training alone was insufficient for behavior change,and other barriers apart from the lack of skills were likely impacting their screening. Training can thus be seen as a first and important step, but combination with multiple implementation strategies (such as supervision or community support) tends to produce better outcomes both on the provider and the patient level (Keurhorst et al., 2015; Rowe et al., 2018).

## Conclusion

The SCALA training programme was well received by the participants and led to more providers conducting alcohol screening in primary health care in Colombia, Mexico and Peru. The training was suitable for different professional roles, but the existing health system structures meant psychologists and doctors were more likely to use the protocol after attending the training, with exact dynamics differing by country. The amount of the training received by the provider was important on the individual level, and the booster sessions were especially important in a context with less institutional support. Overall, our study showed the importance of tailoring the initial training (e.g. adapting sessions based on providers’ existing knowledge) as well as ongoing refinement to better fit the local context.

## Supplemental Material

sj-docx-1-irp-10.1177_26334895221112693 - Supplemental material for Training primary health care providers in Colombia, Mexico and Peru to increase alcohol screening: Mixed-methods process evaluation of implementation strategy Click here for additional data file. Supplemental material, sj-docx-1-irp-10.1177_26334895221112693 for Training primary health care providers in Colombia, Mexico and Peru to increase alcohol screening: Mixed-methods process evaluation of implementation strategy by Daša Kokole, Eva Jané-Llopis, Guillermina Natera Rey, Natalia Bautista Aguilar, Perla Sonia Medina Aguilar, Juliana Mejía-Trujillo, Katherine Mora, Natalia Restrepo, Ines Bustamante, Marina Piazza, Amy O’Donnell, Adriana Solovei, Liesbeth Mercken, Christiane Sybille Schmidt, Hugo Lopez-Pelayo, Silvia Matrai, Fleur Braddick, Antoni Gual, Jürgen Rehm, Peter Anderson and Hein de Vries in Implementation Research and Practice

sj-docx-2-irp-10.1177_26334895221112693 - Supplemental material for Training primary health care providers in Colombia, Mexico and Peru to increase alcohol screening: Mixed-methods process evaluation of implementation strategy Click here for additional data file. Supplemental material, sj-docx-2-irp-10.1177_26334895221112693 for Training primary health care providers in Colombia, Mexico and Peru to increase alcohol screening: Mixed-methods process evaluation of implementation strategy by Daša Kokole, Eva Jané-Llopis, Guillermina Natera Rey, Natalia Bautista Aguilar, Perla Sonia Medina Aguilar, Juliana Mejía-Trujillo, Katherine Mora, Natalia Restrepo, Ines Bustamante, Marina Piazza, Amy O’Donnell, Adriana Solovei, Liesbeth Mercken, Christiane Sybille Schmidt, Hugo Lopez-Pelayo, Silvia Matrai, Fleur Braddick, Antoni Gual, Jürgen Rehm, Peter Anderson and Hein de Vries in Implementation Research and Practice
