# Supplementary material for: Training primary health care providers in Colombia, Mexico and Peru to increase alcohol screening: Mixed-methods process evaluation of implementation strategy
Source: Implement Res Pract. 2022 Jul 15;3:26334895221112693. doi: 10.1177/26334895221112693 (PMC9924276; doi:10.1177/26334895221112693)
Supplement: sj-docx-1-irp-10.1177_26334895221112693 - Supplemental material for Training primary health care providers in Colombia, Mexico and Peru to increase alcohol screening: Mixed-methods process evaluation of implementation strategy [file sj-docx-1-irp-10.1177_26334895221112693.docx]

**Training primary health care providers in Colombia, Mexico and Peru to increase alcohol screening: mixed-methods process evaluation of implementation strategy**

**Supplementary file 1:** Description of training development and adaptation and links to SCALA training materials in English and Spanish

1. **Description of training development and adaptation**

The training development team consisted of Spanish-speaking clinical professionals with previous experience in training development and implementation (also in the field of alcohol screening and brief interventions), combined with academic experts in the field of alcohol and depression. Additionally, the local partners from the three participating countries (Colombia, Mexico and Peru) participated in tailoring the programme to the PHC setting in the participating municipalities.

The training package consisted of 4 products: the training manual, handouts and materials (including evaluation questionnaire), the training course presentations, the training modelling videos, and the TNT (training new trainers) sessions (including slide deck, training materials as above, 2-day in-person course, follow-up ‘reminder’ videos). The general content & structure of the training sessions (presentations and manual) were developed in English based on elements of the WHO 2017 alcohol brief intervention training manual for primary care and PHEPA 2007 Training Programme on Identification and Brief Interventions; as well as on the SCALA clinical package materials. The written materials were revised in English, translated into Spanish, revised and underwent general tailoring for language differences and any health system differences by the Latin American partners. Five training modelling video scripts were developed through live role-play exercises by the lead clinical professionals on the training team, transcribed and translated for assessment by the academic expert team, and revised to shorten and tailored for local language in consultation with the Latin American project partners. The scripts were used to film the model clinical scenarios with a mixture of Latin American actors (from the three countries in SCALA). Initially, the project aim was to only develop and evaluate the standard training on the standard clinical package (in combination with municipal support as the other implementation strategy tested in SCALA), but in the preparation phase, it became clear that development and testing of the short training and package will be necessary to ensure the feasibility in the three countries. This resulted in a further two model scenario videos being developed by the same process – scripted from role play, tailored to Latin American Spanish, and filmed with Latin American actors. All modelling videos were used in the TNT course. TNT course took place in Bogota, Colombia in May 2018. The training was conducted by an addiction specialist with several years of experience in implementing brief interventions and training delivery (also a member of the training development team). Over two full days, the participants (future trainers) experienced the training sessions themselves – the trainer delivered the training unit by unit, followed by discussion and reflection by the whole group. A brief pre-post evaluation showed high satisfaction with the training and increased self-rated knowledge and self-efficacy of the future trainers.

After the TNT course, the materials were revised based on the comments and outcomes of the course sessions (the training team noted areas of difficulty and partners gave feedback on the materials), and training packages for each country, standard and short versions were finalised and made available. To support the on-site training sessions, a series of 3 'refresher' videos were created to highlight hey take-home points from the TNT sessions in the participants own words, with reinforcement of key messages and learning points from the TNT trainer. Further individual tailoring by each trainer was encouraged and facilitated to make their sessions to health professionals as fluid, acceptable and relevant as possible, and to adapt to different constraints in the different intervention sites.

1. **Links to SCALA training materials**
2. *English language materials*

### SHORT FORM TRAINING - ARMS 2 & 3 - EN

[SCALA training manual. Short version. EN](https://www.scalaproject.eu/images/00_EN_SCALA_Training_Manual__Annexes_Short_May_2019.pdf)

[SCALA Training package Session 1. Short version. EN.](https://www.scalaproject.eu/images/01_EN_SCALA_Training_Session_1_slides__notes_short_May_2019.pdf)

[SCALA Training package. Booster session. Short version. EN.](https://www.scalaproject.eu/images/03_EN_SCALA_Booster_Session_slides__notes_short_May_2019.pdf)

### STANDARD FORM TRAINING - ARM 4 - EN

[SCALA training manual. Standard version. EN.](https://www.scalaproject.eu/images/00_EN_SCALA_Training_Manual__Annexes_Long_May_2019.pdf)

[SCALA Training package Session 1. Standard version. EN.](https://www.scalaproject.eu/images/01_EN_SCALA_Training_Session_1_slides__notes_long_May_2019.pdf)

[SCALA Training package Session 2. Standard version. EN..](https://www.scalaproject.eu/images/02_EN_SCALA_Training_Session_2_slides__notes_long_May_2019.pdf)

[SCALA Training package. Booster session. Standard version. EN.](https://www.scalaproject.eu/images/03_EN_SCALA_Booster_Session_slides__notes_long_May_2019.pdf)

1. *Spanish language materials*

### CAPACITACIÓN PROVEEDORES VERSIÓN CORTA - BRAZOS 2 & 3 - ES

[Manual de Formación & Anexos. Version corta. ES.](https://www.scalaproject.eu/images/00_ES_SCALA_Manual_de_Formaci%C3%B3n__Anexos_corto_mayo_2019.pdf)

[Capacitación Sesión 1 diapos & notas. Version corta. ES.](https://www.scalaproject.eu/images/01_ES_SCALA_Capacitaci%C3%B3n_Sesi%C3%B3n_1_diapos__notas_corta_mayo_2019.pdf)

[Sesión Refuerzo diapos & notas. Vesion corta. ES.](https://www.scalaproject.eu/images/03_ES_SCALA_Sesi%C3%B3n_Refuerzo_diapos__notas_corta_mayo_2019.pdf)

### CAPACITACIÓN PROVEEDORES VERSIÓN ESTÁNDAR - BRAZO 4 - ES

[Manual de Formación & Anexos. Version estándar. ES.](https://www.scalaproject.eu/images/00_ES_SCALA_Manual_de_Formaci%C3%B3n__Anexos_larga_mayo_2019.pdf)

[Capacitación Sesión 1 diapos & notas. Version estándar. ES.](https://www.scalaproject.eu/images/01_ES_SCALA_Capacitaci%C3%B3n_Sesi%C3%B3n_1_diapos__notas_larga_mayo_2019.pdf)

[Capacitación Sesión 2 diapos & notas. Version estándar. ES.](https://www.scalaproject.eu/images/02_ES_SCALA_Capacitaci%C3%B3n_Sesi%C3%B3n_2_diapos__notas_larga_mayo_2019.pdf)

[Sesiones de Refuerzo diapos & notas. Version estándar. ES.](https://www.scalaproject.eu/images/03_ES_SCALA_Sesi%C3%B3n_Refuerzo_diapos__notas_larga_mayo_2019.pdf)

### CAPACITACIÓN DE FORMADORES (TNT) - VERSIONES CORTA Y ESTÁNDAR - ES

**Materiales de prepararación**

[Agenda final.](https://www.scalaproject.eu/images/01_SC_TNT_Agenda_final.pdf)

[Introducción a la Capacitación.](https://www.scalaproject.eu/images/02_SCALA_TNT_-_Introducci%C3%B3n_a_la_Capacitaci%C3%B3n_TNT_SCALA.pdf)

[Manual de entrenamiento. Versión corta.](https://www.scalaproject.eu/images/03_SCALA_TNT_Manual_de_entrenamiento_versi%C3%B3n_corta.pdf)

[Manual de entrenamiento. Versión estándar.](https://www.scalaproject.eu/images/03_SCALA_TNT_Manual_de_entrenamiento_versi%C3%B3n_larga.pdf)

[Guia para proveedores. Versión corta.](https://www.scalaproject.eu/images/04_SCALA_TNT_Guia_para_proveedores_versi%C3%B3n_corta.pdf)

[Guia para proveedores. Versión estándar](https://www.scalaproject.eu/images/04_SCALA_TNT_Guia_para_proveedores_versi%C3%B3n_larga.pdf)

[Guia pacientes alc. Versión corta y estándar](https://www.scalaproject.eu/images/05_SCALA_TNT_guia_pacientes_alc_versi%C3%B3n_corta_y_larga.pdf)

[Consejería Breve pacientes alc. Versión corta y estándar](https://www.scalaproject.eu/images/06_SCALA_TNT_Consejer%C3%ADa_Breve_pacientes_alc_versi%C3%B3n_corta_y_larga.pdf)

[Info pacientes depresión.](https://www.scalaproject.eu/images/07_SCALA_TNT_info_pacientes_depresi%C3%B3n.pdf)

1. *Videos overview (all in Spanish)*

| *Video* | *Content covered* | *Video URL* | *Session* |
| --- | --- | --- | --- |
| Sofia – Video A | Screening Alc- / Dep | <https://youtu.be/hrtuQI0uZ7U> | Short |
| Juan – Video B | Screening Alc+ / Dep- | <https://youtu.be/6Gl_Cp0lAmE> | Short |
| Javier – Video C | BI for alcohol | <https://youtu.be/Q7sfR0nkZwU> | Short |
| Pedro – Video 1a | Screening Alc+ / Dep- | <https://youtu.be/cQ6uJwrDU0M> | Standard 1 |
| Paola – Video 2a | Screening Alc+ / Dep+ | <https://youtu.be/e5cfXembmc8> | Standard 1 |
| Pedro – Video 1b | BI for alcohol | <https://youtu.be/pDDGCeLnuYk> | Standard 1 |
| Paola – Video 2b | BI for alcohol + depression | <https://youtu.be/dEjA32_z5Co> | Standard 2 |
| Ana Maria – Video 3 | Referral for alcohol problems and co-morbid depression | <https://youtu.be/Qv4UZHL3vQ8> | Standard 2 |
