# Supplementary material for: Training primary health care providers in Colombia, Mexico and Peru to increase alcohol screening: Mixed-methods process evaluation of implementation strategy
Source: Implement Res Pract. 2022 Jul 15;3:26334895221112693. doi: 10.1177/26334895221112693 (PMC9924276; doi:10.1177/26334895221112693)
Supplement: sj-docx-2-irp-10.1177_26334895221112693 - Supplemental material for Training primary health care providers in Colombia, Mexico and Peru to increase alcohol screening: Mixed-methods process evaluation of implementation strategy [file sj-docx-2-irp-10.1177_26334895221112693.docx]

**Training primary health care providers in Colombia, Mexico and Peru to increase alcohol screening: mixed-methods process evaluation of implementation strategy**

**Supplementary file 2: Supplementary Tables**

*Table S1*: Sample description (providers attending at least one training up to month 5)

*Table S2:* Reach and dose of the training - overall, by country and by arm

*Table S3:* Comparison of eligible providers attending and not attending training by age, gender and professional role

*Table S4*: Post training questionnaire response - satisfaction with aspects of training, by country

*Table S5*: Post training questionnaire response - perceived utility with aspects of training, by country

*Table S6:* Post training questionnaire response - satisfaction with aspects of training, by arm

*Table S7:* Post training questionnaire response - perceived utility with aspects of training, by arm

*Table S8*: Summary of open answers to post-training questionnaire - comments regarding training and suggestions for improvements

*Table S9*: Relationship between implementation factors and outcome; comparison between screeners and non-screeners overall and by country

*Table S10:* Relationship between participant response and outcome; comparison between screeners and non-screeners overall and by country

*Table S11:* Relationship between contextual factors and outcome; comparison between screeners and non-screeners overall and by country

Table S1: Sample description (providers attending at least one training up to month 5)

| ***Sample description*** | | | | |
| --- | --- | --- | --- | --- |
|  | Colombia (N=67, 19.0%) | Mexico (N=139, 39.5%) | Peru (N=146, 41%) | Overall (N=352, 100%) |
|  | M (SD) / % | M (SD) / % | M (SD) / % | M (SD) / % |
| *Gender*  Female  Male | 76.1  23.9 | 68.3  31.7 | 84.9  15.1 | 76.7  23.3 |
| *Profession^a^*  Doctor  Nurse  Psychologist  Other^a^ | 43.3  23.9  1.5  31.3 | 58.3  8.6  8.6  23.0 | 17.8  13.7  13.7  54.8 | 38.6  13.6  9.4  37.8 |
| *Age^b^* | 32.16 (9.84) | 35.59 (12.94) | 44.42 (10.59) | 38.69 (12.48) |
| *Arm*  2 – short training only  3 – short training + municipal support  4 – standard training + municipal support | 43.3  31.3  25.4 | 33.1  36.7  30.2 | 46.6  23.3  30.1 | 40.6  30.1  29.3 |
| *Did screening in baseline period* | 22.4 | 36.7 | 5.5 | 21.0 |
| *Did screening in implementation period* | 76.1 | 52.5 | 33.6 | 49.1 |
| *Attended at least 1 training session* | 100.0 | 100.0 | 100.0 | 100.0 |

^a^ missing data for two providers in Mexico

^b^ missing data for three providers in Colombia, four in Mexico

*Table S2:* Reach and dose of the training - overall, by country and by arm

|  |  | Reach | | | | | | | | | | | | Dose delivered | | Dose received | | | | | | | |
| --- | --- | --- | --- | --- | --- | --- | --- | --- | --- | --- | --- | --- | --- | --- | --- | --- | --- | --- | --- | --- | --- | --- | --- |
|  |  | Attendance T1 | | | Attendance T2 | | | Attendance booster session | | | Attendance at least 1 session | | | Hours of training offered | Number of sessions offered (incl. booster) | Number of hours in training | | | | Number of sessions attended | | | |
|  |  | N attended | N eligible | % reached | N attended | N eligible | % reached | N attended | N eligible | % reached | N attended | N eligible | % reached |  |  | Min | Max | M | SD | One session | Two sessions | Three sessions |  |
| Total | | 309 | 452 | 68.4% | 69 | 116 | 59.5% | 140 | 300 | 46.7% | 352 | 487 | 72.3% |  |  | 1.0 | 6.0 | 2.7 | 1.2 | 58.5% | 36.1% | 5.4% |  |
| Colombia | | 56 | 66 | 84.8% | 0 | 0 |  | 45 | 55 | 81.8% | 67 | 75 | 89.3% |  |  | 1.5 | 4.0 | 2.7 | 1.0 | 49.3% | 50.7% |  |  |
| Mexico | | 112 | 188 | 59.6% | 38 | 51 | 74.5% | 43 | 111 | 38.7% | 139 | 214 | 65.0% |  |  | 1.0 | 5.0 | 2.2 | 0.8 | 63.3% | 34.5% | 2.2% |  |
| Peru | | 141 | 198 | 71.2% | 31 | 65 | 47.7% | 52 | 134 | 38.8% | 146 | 198 | 73.7% |  |  | 2.0 | 6.0 | 3.1 | 1.4 | 58.2% | 30.8% | 11.0% |  |
| Arm 2 | | 120 | 167 | 71.9% |  |  |  | 61 | 141 | 43.3% | 143 | 193 | 74.1% |  |  | 1.0 | 4.0 | 2.3 | 0.9 | 74.1% | 25.9% |  |  |
|  | Colombia | 20 | 27 | 74.1% |  |  |  | 19 | 25 | 76.0% | 29 | 35 | 82.9% | 3.5 | 2 | 1.5 | 3.5 | 2.3 | 0.9 | 65.5% | 34.5% |  |  |
|  | Mexico | 34 | 56 | 60.7% |  |  |  | 20 | 52 | 38.5% | 46 | 74 | 62.2% | 3.0 | 2 | 1.0 | 3.0 | 1.9 | 0.7 | 82.6% | 17.4% |  |  |
|  | Peru | 66 | 84 | 78.6% |  |  |  | 22 | 64 | 34.4% | 68 | 84 | 81.0% | 4.0 | 2 | 2.0 | 4.0 | 2.6 | 0.9 | 72.1% | 27.9% |  |  |
| Arm 3 | | 100 | 153 | 65.4% |  |  |  | 41 | 83 | 49.4% | 106 | 160 | 66.3% |  |  | 1.0 | 4.0 | 2.4 | 0.8 | 67.0% | 33.0% |  |  |
|  | Colombia | 21 | 23 | 91.3% |  |  |  | 15 | 17 | 88.2% | 21 | 23 | 91.3% | 3.5 | 2 | 1.5 | 3.5 | 2.9 | 0.9 | 28.6% | 71.4% |  |  |
|  | Mexico | 45 | 81 | 55.6% |  |  |  | 16 | 42 | 38.1% | 51 | 88 | 58.0% | 3.0 | 2 | 1.0 | 3.0 | 2.1 | 0.6 | 80.4% | 19.6% |  |  |
|  | Peru | 34 | 49 | 69.4% |  |  |  | 10 | 24 | 41.7% | 34 | 49 | 69.4% | 4.0 | 2 | 2.0 | 4.0 | 2.6 | 0.9 | 70.6% | 29.4% |  |  |
| Arm 4 | | 89 | 132 | 67.4% | 69 | 116 | 59.5% | 38 | 76 | 50.0% | 103 | 134 | 76.9% |  |  | 1.0 | 6.0 | 3.4 | 1.4 | 28.2% | 53.4% | 18.4% |  |
|  | Colombia | 15 | 16 | 93.8% | 0 | 0 |  | 11 | 13 | 84.6% | 17 | 17 | 100.0% | 4.0 | 2 | 2.0 | 4.0 | 3.1 | 1.0 | 47.1% | 52.9% |  |  |
|  | Mexico | 33 | 51 | 64.7% | 38 | 51 | 74.5% | 7 | 17 | 41.2% | 42 | 52 | 80.8% | 5.0 | 3 | 1.0 | 5.0 | 2.8 | 1.0 | 21.4% | 71.4% | 7.1% |  |
|  | Peru | 41 | 65 | 63.1% | 31 | 65 | 47.7% | 20 | 46 | 43.5% | 44 | 65 | 67.7% | 6.0 | 3 | 2.0 | 6.0 | 4.2 | 1.6 | 27.3% | 36.4% | 36.4% |  |

*Table S3*: Comparison of eligible providers attending and not attending training by age, gender and professional role

|  |  | N | | Age | | | | Gender | | | Professional role | | |
| --- | --- | --- | --- | --- | --- | --- | --- | --- | --- | --- | --- | --- | --- |
|  |  | Attending | Non-attending | M(SD) - attending | M(SD) - non attending | Mann-U | p | % attending | Chi square | p | % attending | Chi square | p |
| Total | | 352 | 135 | 38.7 (12.5) | 40.0 (12.4) | 21764 | 0.320 | 74.1% eligible females vs. 65.1% eligible males | 4.40 | **0.036** | 67.7% of all eligible doctors, 71.6% nurses, 82.5% psychologists, 75.1% others | 4.95 | 0.176 |
| Colombia | | 67 | 8 | 32.2 (9.8) | 37.9 (14.8) | 207.5 | 0.384 | 86.4% eligible females vs. 100% eligible males | 2.43 | 0.119 | 93.5% of all eligible doctors, 94.1% nurses, 100% psychologists, 80.8% others | 3.11 | 0.375 |
| Mexico | | 139 | 75 | 35.6 (12.9) | 36.6 (11.9) | 4670.5 | 0.437 | 65.5% eligible females vs. 63.8% eligible males | 0.06 | 0.802 | 63.3% of all eligible doctors, 52.2% nurses, 100% psychologists, 65.2% others | 8.24 | **0.041** |
| Peru | | 146 | 52 | 44.4 (10.6) | 45.1 (11.1) | 3647 | 0.674 | 79.0% eligible females vs. 53.7% eligible males | 10.77 | **0.001** | 61.9% of all eligible doctors, 74.1% nurses, 74.1% psychologists, 78.4% others | 4.20 | 0.241 |
| Arm 2 | | 143 | 50 | 42.2 (12.7) | 38.6 (12.7) | 4126 | 0.073 | 74.8% eligible females vs. 71.7% eligible males | 0.17 | 0.676 | 69.4% of all eligible doctors, 72.4% nurses, 85.7% psychologists, 76.9% others | 2.16 | 0.539 |
|  | Colombia^a^ | 29 | 6 | 29.9 (8.8) | 35.8 (13.1) | 64 | 0.388 | 79.3% eligible females vs. 100% eligible males | 1.50 | 0.221 | 87.5% of all eligible doctors, 100% nurses, 73.7% others | 2.90 | 0.234 |
|  | Mexico^b^ | 46 | 28 | 43.5 (13.7) | 36.1 (11.0) | 846.5 | **0.014** | 61.7% eligible females vs. 63.0% eligible males | 0.01 | 0.914 | 64.4% of all eligible doctors, 45.5% nurses, 100% psychologists, 50.0% others | 5.10 | 0.165 |
|  | Peru | 68 | 16 | 46.4 (10.1) | 44.0 (13.1) | 615.5 | 0.415 | 81.7% eligible females vs. 76.9% eligible males | 0.17 | 0.687 | 75.0% of all eligible doctors, 80% nurses, 77.8% psychologists, 83.7% others | 0.67 | 0.881 |
| Arm 3 | | 106 | 54 | 36.2 (11.8) | 40.5 (13.6) | 2293 | 0.085 | 71.0% eligible females vs. 50% eligible males | 5.49 | **0.019** | 57.6% of all eligible doctors, 70.4% nurses, 84.6% psychologists, 70.4% others | 4.80 | 0.187 |
|  | Colombia | 21 | 2 | 34.7 (10.6) | 44.0 (24.0) | 14 | 0.506 | 88.9% eligible females vs. 100% eligible males | 0.61 | 0.435 | 93.3% of all eligible doctors, 80% nurses, 100% others | 1.17 | 0.558 |
|  | Mexico^c^ | 51 | 37 | 30.9 (9.7) | 36.8 (12.9) | 650 | **0.039** | 59.1% eligible females vs. 54.5% eligible males | 0.14 | 0.708 | 48.8% of all eligible doctors, 58.3% nurses, 100% psychologists,65.6% others | 4.37 | 0.225 |
|  | Peru | 34 | 15 | 45.0 (10.3) | 48.8 (10.8) | 187.5 | 0.143 | 82.5% eligible females vs. 11.1% eligible males | 17.63 | **<0.001** | 40.0% of all eligible doctors, 80.0% nurses, 80.0% psychologists, 73.7% others | 5.291h | 0.152 |
| Arm 4 | | 103 | 31 | 36.3 (11.8) | 41.1 (10.4) | 1114.5 | **0.018** | 80.0% eligible females vs. 70.5% eligible males | 1.51 | 0.218 | 76.2% of all eligible doctors, 72.7% nurses, 76.9% psychologists, 77.8% others | 0.13 | 0.988 |
|  | Colombia | 17 | 0 | Comparison not possible as all eligible providers participated | | | | |  |  |  |  |  |
|  | Mexico^d^ | 42 | 10 | 32.7 (11.6) | 36.8 (11.4) | 157 | 0.253 | 84.4% eligible females vs. 75.0% eligible males | 0.70 | 0.404 | 76.9% of all eligible doctors, 100% psychologists, 85.7% others | 1.37 | 0.503 |
|  | Peru | 44 | 21 | 40.9 (11.0) | 43.2 (9.5) | 389 | 0.305 | 71.7% eligible females vs. 57.9% eligible males | 1.18 | 0.278 | 62.5% of all eligible doctors, 57.1% nurses, 62.5% psychologists, 73.5% others | 1.18 | 0.757 |

^a^ Age data for 1 attending provider missing

^b^ Age data for 1 attending provider missing

^c^ Age data for 2 attending and 1 non attending providers missing

^d^ 1 attending provider missing age data, 2 attending providers missing professional role data

*Table S4:* Post training questionnaire response - satisfaction with aspects of training, by country (COL=Colombia, MEX= Mexico, PER=Peru)

|  |  |  | N | M | SD | Min | Max | Kruskal-Wallis H | Sig | Post-hoc |
| --- | --- | --- | --- | --- | --- | --- | --- | --- | --- | --- |
| Training 1 | Overall experience with the course | COL | 46 | 4.46 | 0.55 | 3 | 5 | 3.40 | 0.182 |  |
|  |  | MEX | 113 | 4.61 | 0.51 | 3 | 5 |  |  |  |
|  |  | PER | 136 | 4.52 | 0.52 | 3 | 5 |  |  |  |
|  |  | Total | 295 | 4.55 | 0.52 | 3 | 5 |  |  |  |
|  | Information received before the course | COL | 46 | 4.02 | 0.61 | 3 | 5 | 7.49 | **0.024** | COL < MEX |
|  |  | MEX | 112 | 4.32 | 0.70 | 2 | 5 |  |  |  |
|  |  | PER | 133 | 4.20 | 0.70 | 2 | 5 |  |  |  |
|  |  | Total | 291 | 4.22 | 0.69 | 2 | 5 |  |  |  |
|  | Location | COL | 46 | 3.98 | 0.77 | 2 | 5 | 15.97 | **<0.001** | COL <MEX, COL< PER |
|  |  | MEX | 112 | 4.47 | 0.66 | 2 | 5 |  |  |  |
|  |  | PER | 135 | 4.36 | 0.65 | 2 | 5 |  |  |  |
|  |  | Total | 293 | 4.34 | 0.69 | 2 | 5 |  |  |  |
|  | Venue | COL | 46 | 3.93 | 0.71 | 2 | 5 | 22.87 | **<0.001** | COL <MEX, COL< PER |
|  |  | MEX | 113 | 4.51 | 0.58 | 3 | 5 |  |  |  |
|  |  | PER | 136 | 4.35 | 0.65 | 3 | 5 |  |  |  |
|  |  | Total | 295 | 4.35 | 0.66 | 2 | 5 |  |  |  |
|  | Duration | COL | 46 | 4.15 | 0.63 | 3 | 5 | 3.88 | 0.144 |  |
|  |  | MEX | 112 | 4.33 | 0.62 | 3 | 5 |  |  |  |
|  |  | PER | 134 | 4.35 | 0.62 | 2 | 5 |  |  |  |
|  |  | Total | 292 | 4.31 | 0.62 | 2 | 5 |  |  |  |
|  | Trainer | COL | 46 | 4.59 | 0.50 | 4 | 5 | 8.48 | **0.014** | COL < MEX |
|  |  | MEX | 113 | 4.80 | 0.40 | 4 | 5 |  |  |  |
|  |  | PER | 136 | 4.66 | 0.49 | 3 | 5 |  |  |  |
|  |  | Total | 295 | 4.70 | 0.47 | 3 | 5 |  |  |  |
|  | My participation | COL | 46 | 4.13 | 0.62 | 3 | 5 | 3.29 | 0.193 |  |
|  |  | MEX | 113 | 4.30 | 0.64 | 3 | 5 |  |  |  |
|  |  | PER | 136 | 4.22 | 0.54 | 3 | 5 |  |  |  |
|  |  | Total | 295 | 4.24 | 0.59 | 3 | 5 |  |  |  |
|  | Other participants | COL | 46 | 4.13 | 0.58 | 3 | 5 | 1.92 | 0.382 |  |
|  |  | MEX | 109 | 4.24 | 0.72 | 2 | 5 |  |  |  |
|  |  | PER | 132 | 4.26 | 0.49 | 3 | 5 |  |  |  |
|  |  | Total | 287 | 4.23 | 0.60 | 2 | 5 |  |  |  |
| Training 2 | Overall experience with the course | COL |  |  |  |  |  | 7.28 | **0.007** | MEX < PER |
|  |  | MEX | 24 | 4.33 | 0.48 | 4 | 5 |  |  |  |
|  |  | PER | 33 | 4.70 | 0.47 | 4 | 5 |  |  |  |
|  |  | Total | 57 | 4.54 | 0.50 | 4 | 5 |  |  |  |
|  | Information received before the course | COL | 0 |  |  |  |  | 0.92 | 0.337 |  |
|  |  | MEX | 24 | 4.38 | 0.65 | 3 | 5 |  |  |  |
|  |  | PER | 33 | 4.21 | 0.65 | 3 | 5 |  |  |  |
|  |  | Total | 57 | 4.28 | 0.65 | 3 | 5 |  |  |  |
|  | Location | COL |  |  |  |  |  | 0.06 | 0.808 |  |
|  |  | MEX | 24 | 4.42 | 0.72 | 3 | 5 |  |  |  |
|  |  | PER | 32 | 4.50 | 0.57 | 3 | 5 |  |  |  |
|  |  | Total | 56 | 4.46 | 0.63 | 3 | 5 |  |  |  |
|  | Venue | COL |  |  |  |  |  | 0.13 | 0.719 |  |
|  |  | MEX | 24 | 4.46 | 0.59 | 3 | 5 |  |  |  |
|  |  | PER | 33 | 4.52 | 0.57 | 3 | 5 |  |  |  |
|  |  | Total | 57 | 4.49 | 0.57 | 3 | 5 |  |  |  |
|  | Duration | COL |  |  |  |  |  | 3.77 | 0.052 | MEX < PER |
|  |  | MEX | 23 | 3.87 | 1.06 | 2 | 5 |  |  |  |
|  |  | PER | 33 | 4.42 | 0.61 | 3 | 5 |  |  |  |
|  |  | Total | 56 | 4.20 | 0.86 | 2 | 5 |  |  |  |
|  | Trainer | COL |  |  |  |  |  | 0.01 | 0.931 |  |
|  |  | MEX | 24 | 4.63 | 0.49 | 4 | 5 |  |  |  |
|  |  | PER | 33 | 4.64 | 0.49 | 4 | 5 |  |  |  |
|  |  | Total | 57 | 4.63 | 0.49 | 4 | 5 |  |  |  |
|  | My participation | COL |  |  |  |  |  | 2.58 | 0.108 |  |
|  |  | MEX | 24 | 4.04 | 0.69 | 3 | 5 |  |  |  |
|  |  | PER | 33 | 4.33 | 0.60 | 3 | 5 |  |  |  |
|  |  | Total | 57 | 4.21 | 0.65 | 3 | 5 |  |  |  |
|  | Other participants | COL |  |  |  |  |  | 0.66 | 0.416 |  |
|  |  | MEX | 23 | 4.13 | 0.69 | 3 | 5 |  |  |  |
|  |  | PER | 31 | 4.29 | 0.53 | 3 | 5 |  |  |  |
|  |  | Total | 54 | 4.22 | 0.60 | 3 | 5 |  |  |  |
| Booster session | Overall experience | COL | 36 | 4.39 | 0.64 | 3 | 5 | 4.76 | 0.093 |  |
|  |  | MEX | 21 | 4.67 | 0.58 | 3 | 5 |  |  |  |
|  |  | PER | 51 | 4.61 | 0.70 | 1 | 5 |  |  |  |
|  |  | Total | 108 | 4.55 | 0.66 | 1 | 5 |  |  |  |

*Table S5:* Post training questionnaire response – perceived utility with aspects of training, by country (COL=Colombia, MEX= Mexico, PER=Peru)

|  |  |  | N | M | SD | Min | Max | Kruskal-Wallis H | Sig | Post-hoc |
| --- | --- | --- | --- | --- | --- | --- | --- | --- | --- | --- |
| Training 1 | Overall training 1 | COL | 46 | 4.59 | 0.54 | 3 | 5 | 1.57 | 0.457 |  |
|  |  | MEX | 105 | 4.70 | 0.48 | 3 | 5 |  |  |  |
|  |  | PER | 135 | 4.63 | 0.54 | 2 | 5 |  |  |  |
|  |  | Total | 286 | 4.65 | 0.52 | 2 | 5 |  |  |  |
|  | Information on impact of alcohol and costs of alcohol use | COL | 46 | 4.46 | 0.62 | 3 | 5 | 5.48 | 0.064 |  |
|  |  | MEX | 98 | 4.59 | 0.53 | 3 | 5 |  |  |  |
|  |  | PER | 134 | 4.39 | 0.65 | 3 | 5 |  |  |  |
|  |  | Total | 278 | 4.47 | 0.61 | 3 | 5 |  |  |  |
|  | Discussion on attitudes to alcohol | COL | 46 | 4.39 | 0.54 | 3 | 5 | 1.90 | 0.387 |  |
|  |  | MEX | 98 | 4.51 | 0.58 | 3 | 5 |  |  |  |
|  |  | PER | 134 | 4.46 | 0.58 | 2 | 5 |  |  |  |
|  |  | Total | 278 | 4.46 | 0.57 | 2 | 5 |  |  |  |
|  | Phrases to start the discussion with the patient | COL | 46 | 4.41 | 0.50 | 4 | 5 | 1.40 | 0.496 |  |
|  |  | MEX | 97 | 4.48 | 0.58 | 3 | 5 |  |  |  |
|  |  | PER | 133 | 4.51 | 0.52 | 3 | 5 |  |  |  |
|  |  | Total | 276 | 4.49 | 0.54 | 3 | 5 |  |  |  |
|  | Presentation of screening criteria for SCALA | COL | 46 | 4.48 | 0.51 | 4 | 5 | 1.51 | 0.471 |  |
|  |  | MEX | 98 | 4.53 | 0.56 | 3 | 5 |  |  |  |
|  |  | PER | 134 | 4.45 | 0.56 | 3 | 5 |  |  |  |
|  |  | Total | 278 | 4.48 | 0.55 | 3 | 5 |  |  |  |
|  | Role play to practice screening | COL | 46 | 4.30 | 0.55 | 3 | 5 | 5.75 | 0.057 |  |
|  |  | MEX | 98 | 4.50 | 0.65 | 3 | 5 |  |  |  |
|  |  | PER | 134 | 4.51 | 0.54 | 3 | 5 |  |  |  |
|  |  | Total | 278 | 4.47 | 0.59 | 3 | 5 |  |  |  |
|  | Presentation of steps of brief intervention | COL | 46 | 4.52 | 0.51 | 4 | 5 | 17.62 | **<0.001** | PER < MEX |
|  |  | MEX | 98 | 4.71 | 0.54 | 3 | 5 |  |  |  |
|  |  | PER | 134 | 4.43 | 0.58 | 3 | 5 |  |  |  |
|  |  | Total | 278 | 4.54 | 0.57 | 3 | 5 |  |  |  |
|  | Role play to practice delivering brief intervention | COL | 46 | 4.41 | 0.65 | 3 | 5 | 0.76 | 0.683 |  |
|  |  | MEX | 98 | 4.48 | 0.69 | 3 | 5 |  |  |  |
|  |  | PER | 134 | 4.48 | 0.54 | 3 | 5 |  |  |  |
|  |  | Total | 278 | 4.47 | 0.62 | 3 | 5 |  |  |  |
| Training 2 | Overall training 2 | COL |  |  |  |  |  | 4.64 | **0.031** | MEX < PER |
|  |  | MEX | 14 | 4.43 | 0.51 | 4 | 5 |  |  |  |
|  |  | PER | 33 | 4.76 | 0.44 | 4 | 5 |  |  |  |
|  |  | Total | 47 | 4.66 | 0.48 | 4 | 5 |  |  |  |
|  | Role playing delivering brief intervention for alcohol with comorbid depressive symptoms | COL | 13 | 4.54 | 0.52 | 4 | 5 | 1.85 | 0.397 |  |
|  |  | MEX | 21 | 4.62 | 0.50 | 4 | 5 |  |  |  |
|  |  | PER | 34 | 4.74 | 0.45 | 4 | 5 |  |  |  |
|  |  | Total | 68 | 4.66 | 0.48 | 4 | 5 |  |  |  |
|  | Learning about referral | COL | 13 | 4.23 | 0.60 | 3 | 5 | 8.52 | **0.014** | COL < PER |
|  |  | MEX | 21 | 4.29 | 0.72 | 3 | 5 |  |  |  |
|  |  | PER | 34 | 4.71 | 0.46 | 4 | 5 |  |  |  |
|  |  | Total | 68 | 4.49 | 0.61 | 3 | 5 |  |  |  |
|  | Role playing for referring patients | COL | 13 | 4.31 | 0.48 | 4 | 5 | 10.48 | **0.005** | COL < PER, MEX < PER |
|  |  | MEX | 21 | 4.29 | 0.64 | 3 | 5 |  |  |  |
|  |  | PER | 34 | 4.74 | 0.45 | 4 | 5 |  |  |  |
|  |  | Total | 68 | 4.51 | 0.56 | 3 | 5 |  |  |  |
|  | Learning about treatment options when referral is not possible | COL | 13 | 4.31 | 0.48 | 4 | 5 | 15.50 | **<0.001** | COL < PER, MEX < PER |
|  |  | MEX | 21 | 4.24 | 0.62 | 3 | 5 |  |  |  |
|  |  | PER | 34 | 4.79 | 0.41 | 4 | 5 |  |  |  |
|  |  | Total | 68 | 4.53 | 0.56 | 3 | 5 |  |  |  |
| Booster session | Overall session | COL | 36 | 4.47 | 0.65 | 3 | 5 | 2.91 | 0.234 |  |
|  |  | MEX | 21 | 4.67 | 0.66 | 3 | 5 |  |  |  |
|  |  | PER | 51 | 4.69 | 0.47 | 4 | 5 |  |  |  |
|  |  | Total | 108 | 4.61 | 0.58 | 3 | 5 |  |  |  |
|  | Exchange of experience with other providers | COL | 36 | 4.33 | 0.79 | 2 | 5 | 1.63 | 0.443 |  |
|  |  | MEX | 21 | 4.52 | 0.75 | 3 | 5 |  |  |  |
|  |  | PER | 51 | 4.41 | 0.50 | 4 | 5 |  |  |  |
|  |  | Total | 108 | 4.41 | 0.66 | 2 | 5 |  |  |  |
|  | Getting practical solutions to problems | COL | 36 | 4.31 | 0.75 | 3 | 5 | 1.59 | 0.451 |  |
|  |  | MEX | 21 | 4.43 | 0.75 | 3 | 5 |  |  |  |
|  |  | PER | 50 | 4.54 | 0.50 | 4 | 5 |  |  |  |
|  |  | Total | 107 | 4.44 | 0.65 | 3 | 5 |  |  |  |

Table S6: Post training questionnaire response - satisfaction with aspects of training, by arm

|  |  |  | N | M | SD | Min | Max | Kruskal-Wallis H | Sig | Post-hoc |
| --- | --- | --- | --- | --- | --- | --- | --- | --- | --- | --- |
| Training 1 | Overall experience with the course | Arm 2 | 111 | 4.62 | 0.51 | 3 | 5 | 4.04 | 0.133 |  |
|  |  | Arm 3 | 96 | 4.49 | 0.52 | 3 | 5 |  |  |  |
|  |  | Arm 4 | 88 | 4.51 | 0.53 | 3 | 5 |  |  |  |
|  |  | Total | 295 | 4.55 | 0.52 | 3 | 5 |  |  |  |
|  | Information received before the course | Arm 2 | 109 | 4.31 | 0.66 | 3 | 5 | 4.44 | 0.109 |  |
|  |  | Arm 3 | 95 | 4.08 | 0.78 | 2 | 5 |  |  |  |
|  |  | Arm 4 | 87 | 4.25 | 0.61 | 3 | 5 |  |  |  |
|  |  | Total | 291 | 4.22 | 0.69 | 2 | 5 |  |  |  |
|  | Location | Arm 2 | 109 | 4.39 | 0.71 | 2 | 5 | 4.22 | 0.121 |  |
|  |  | Arm 3 | 96 | 4.23 | 0.72 | 2 | 5 |  |  |  |
|  |  | Arm 4 | 88 | 4.42 | 0.64 | 3 | 5 |  |  |  |
|  |  | Total | 293 | 4.34 | 0.69 | 2 | 5 |  |  |  |
|  | Venue | Arm 2 | 111 | 4.40 | 0.65 | 2 | 5 | 6.00 | **0.050** | Arm 3 < Arm 4. insignificant after adjusting for multiple testing |
|  |  | Arm 3 | 96 | 4.21 | 0.69 | 2 | 5 |  |  |  |
|  |  | Arm 4 | 88 | 4.43 | 0.62 | 3 | 5 |  |  |  |
|  |  | Total | 295 | 4.35 | 0.66 | 2 | 5 |  |  |  |
|  | Duration | Arm 2 | 111 | 4.45 | 0.53 | 3 | 5 | 10.01 | **0.007** | Arm 3 < Arm 2 |
|  |  | Arm 3 | 93 | 4.16 | 0.63 | 2 | 5 |  |  |  |
|  |  | Arm 4 | 88 | 4.30 | 0.68 | 3 | 5 |  |  |  |
|  |  | Total | 292 | 4.31 | 0.62 | 2 | 5 |  |  |  |
|  | Trainer | Arm 2 | 111 | 4.74 | 0.46 | 3 | 5 | 1.49 | 0.476 |  |
|  |  | Arm 3 | 96 | 4.69 | 0.47 | 4 | 5 |  |  |  |
|  |  | Arm 4 | 88 | 4.67 | 0.47 | 4 | 5 |  |  |  |
|  |  | Total | 295 | 4.70 | 0.47 | 3 | 5 |  |  |  |
|  | My participation | Arm 2 | 111 | 4.21 | 0.52 | 3 | 5 | 2.64 | 0.267 |  |
|  |  | Arm 3 | 96 | 4.20 | 0.64 | 3 | 5 |  |  |  |
|  |  | Arm 4 | 88 | 4.32 | 0.62 | 3 | 5 |  |  |  |
|  |  | Total | 295 | 4.24 | 0.59 | 3 | 5 |  |  |  |
|  | Other participants | Arm 2 | 109 | 4.27 | 0.50 | 3 | 5 | 1.62 | 0.445 |  |
|  |  | Arm 3 | 91 | 4.15 | 0.67 | 2 | 5 |  |  |  |
|  |  | Arm 4 | 87 | 4.26 | 0.64 | 2 | 5 |  |  |  |
|  |  | Total | 287 | 4.23 | 0.60 | 2 | 5 |  |  |  |
| Training 2 | Overall experience with the course | Arm 2 |  |  |  |  |  |  |  |  |
|  |  | Arm 3 |  |  |  |  |  |  |  |  |
|  |  | Arm 4 | 57 | 4.54 | 0.50 | 4 | 5 |  |  |  |
|  |  | Total | 57 | 4.54 | 0.50 | 4 | 5 |  |  |  |
|  | Information received before the course | Arm 2 |  |  |  |  |  |  |  |  |
|  |  | Arm 3 |  |  |  |  |  |  |  |  |
|  |  | Arm 4 | 57 | 4.28 | 0.65 | 3 | 5 |  |  |  |
|  |  | Total | 57 | 4.28 | 0.65 | 3 | 5 |  |  |  |
|  | Location | Arm 2 |  |  |  |  |  |  |  |  |
|  |  | Arm 3 |  |  |  |  |  |  |  |  |
|  |  | Arm 4 | 56 | 4.46 | 0.63 | 3 | 5 |  |  |  |
|  |  | Total | 56 | 4.46 | 0.63 | 3 | 5 |  |  |  |
|  | Venue | Arm 2 |  |  |  |  |  |  |  |  |
|  |  | Arm 3 |  |  |  |  |  |  |  |  |
|  |  | Arm 4 | 57 | 4.49 | 0.57 | 3 | 5 |  |  |  |
|  |  | Total | 57 | 4.49 | 0.57 | 3 | 5 |  |  |  |
|  | Duration | Arm 2 |  |  |  |  |  |  |  |  |
|  |  | Arm 3 |  |  |  |  |  |  |  |  |
|  |  | Arm 4 | 56 | 4.2 | 0.86 | 2 | 5 |  |  |  |
|  |  | Total | 56 | 4.2 | 0.86 | 2 | 5 |  |  |  |
|  | Trainer | Arm 2 |  |  |  |  |  |  |  |  |
|  |  | Arm 3 |  |  |  |  |  |  |  |  |
|  |  | Arm 4 | 57 | 4.63 | 0.49 | 4 | 5 |  |  |  |
|  |  | Total | 57 | 4.63 | 0.49 | 4 | 5 |  |  |  |
|  | My participation | Arm 2 |  |  |  |  |  |  |  |  |
|  |  | Arm 3 |  |  |  |  |  |  |  |  |
|  |  | Arm 4 | 57 | 4.21 | 0.65 | 3 | 5 |  |  |  |
|  |  | Total | 57 | 4.21 | 0.65 | 3 | 5 |  |  |  |
|  | Other participants | Arm 2 |  |  |  |  |  |  |  |  |
|  |  | Arm 3 |  |  |  |  |  |  |  |  |
|  |  | Arm 4 | 54 | 4.22 | 0.60 | 3 | 5 |  |  |  |
|  |  | Total | 54 | 4.22 | 0.60 | 3 | 5 |  |  |  |
| Booster session | Overall experience | Arm 2 | 56 | 4.54 | 0.63 | 3 | 5 | 1.78 | 0.410 |  |
|  |  | Arm 3 | 19 | 4.63 | 0.60 | 3 | 5 |  |  |  |
|  |  | Arm 4 | 33 | 4.73 | 0.45 | 4 | 5 |  |  |  |
|  |  | Total | 108 | 4.61 | 0.58 | 3 | 5 |  |  |  |

*Table S7:* Post training questionnaire response – perceived utility of aspects of training, by arm

|  |  |  | N | M | SD | Min | Max | Kruskal-Wallis H | Sig | Post-hoc |
| --- | --- | --- | --- | --- | --- | --- | --- | --- | --- | --- |
| Training 1 | Overall training 1 | Arm 2 | 109 | 4.66 | 0.53 | 2 | 5 | 0.21 | 0.899 |  |
|  |  | Arm 3 | 95 | 4.63 | 0.53 | 3 | 5 |  |  |  |
|  |  | Arm 4 | 82 | 4.65 | 0.51 | 3 | 5 |  |  |  |
|  |  | Total | 286 | 4.65 | 0.52 | 2 | 5 |  |  |  |
|  | Information on impact of alcohol and costs of alcohol use | Arm 2 | 109 | 4.43 | 0.66 | 3 | 5 | 3.59 | 0.166 |  |
|  |  | Arm 3 | 95 | 4.58 | 0.52 | 3 | 5 |  |  |  |
|  |  | Arm 4 | 74 | 4.39 | 0.64 | 3 | 5 |  |  |  |
|  |  | Total | 278 | 4.47 | 0.61 | 3 | 5 |  |  |  |
|  | Discussion on attitudes to alcohol | Arm 2 | 110 | 4.49 | 0.59 | 2 | 5 | 1.08 | 0.584 |  |
|  |  | Arm 3 | 94 | 4.48 | 0.54 | 3 | 5 |  |  |  |
|  |  | Arm 4 | 74 | 4.41 | 0.59 | 3 | 5 |  |  |  |
|  |  | Total | 278 | 4.46 | 0.57 | 2 | 5 |  |  |  |
|  | Phrases to start the discussion with the patient | Arm 2 | 108 | 4.49 | 0.52 | 3 | 5 | 0.22 | 0.895 |  |
|  |  | Arm 3 | 94 | 4.50 | 0.54 | 3 | 5 |  |  |  |
|  |  | Arm 4 | 74 | 4.46 | 0.55 | 3 | 5 |  |  |  |
|  |  | Total | 276 | 4.49 | 0.54 | 3 | 5 |  |  |  |
|  | Presentation of screening criteria for SCALA | Arm 2 | 109 | 4.45 | 0.55 | 3 | 5 | 0.83 | 0.661 |  |
|  |  | Arm 3 | 95 | 4.48 | 0.56 | 3 | 5 |  |  |  |
|  |  | Arm 4 | 74 | 4.53 | 0.53 | 3 | 5 |  |  |  |
|  |  | Total | 278 | 4.48 | 0.55 | 3 | 5 |  |  |  |
|  | Role play to practice screening | Arm 2 | 110 | 4.54 | 0.54 | 3 | 5 | 1.44 | 0.487 |  |
|  |  | Arm 3 | 94 | 4.43 | 0.63 | 3 | 5 |  |  |  |
|  |  | Arm 4 | 74 | 4.45 | 0.06 | 3 | 5 |  |  |  |
|  |  | Total | 278 | 4.47 | 0.59 | 3 | 5 |  |  |  |
|  | Presentation of steps of brief intervention | Arm 2 | 110 | 4.50 | 0.57 | 3 | 5 | 1.28 | 0.526 |  |
|  |  | Arm 3 | 94 | 4.57 | 0.58 | 3 | 5 |  |  |  |
|  |  | Arm 4 | 74 | 4.57 | 0.55 | 3 | 5 |  |  |  |
|  |  | Total | 278 | 4.54 | 0.57 | 3 | 5 |  |  |  |
|  | Role play to practice delivering brief intervention | Arm 2 | 110 | 4.45 | 0.57 | 3 | 5 | 0.81 | 0.668 |  |
|  |  | Arm 3 | 94 | 4.48 | 0.65 | 3 | 5 |  |  |  |
|  |  | Arm 4 | 74 | 4.49 | 0.65 | 3 | 5 |  |  |  |
|  |  | Total | 278 | 4.47 | 0.62 | 3 | 5 |  |  |  |
| Training 2* | Overall training 2 | Arm 2 |  |  |  |  |  |  |  |  |
|  |  | Arm 3 |  |  |  |  |  |  |  |  |
|  |  | Arm 4 | 47 | 4.66 | 0.48 | 4 | 5 |  |  |  |
|  |  | Total | 47 | 4.66 | 0.48 | 4 | 5 |  |  |  |
|  | Role playing delivering brief intervention for alcohol with comorbid depressive symptoms | Arm 2 |  |  |  |  |  | 3.97 | 0.046 |  |
|  |  | Arm 3 | 2 | 4 | 0 | 4 | 4 |  |  |  |
|  |  | Arm 4 | 66 | 4.68 | 0.47 | 4 | 5 |  |  |  |
|  |  | Total | 68 | 4.66 | 0.48 | 4 | 5 |  |  |  |
|  | Learning about referral | Arm 2 |  |  |  |  |  | 1.85 | 0.174 |  |
|  |  | Arm 3 | 2 | 4 | 0 | 4 | 4 |  |  |  |
|  |  | Arm 4 | 66 | 4.50 | 0.61 | 3 | 5 |  |  |  |
|  |  | Total | 68 | 4.49 | 0.61 | 3 | 5 |  |  |  |
|  | Role playing for referring patients | Arm 2 |  |  |  |  |  | 2.12 | 0.145 |  |
|  |  | Arm 3 | 2 | 4 | 0 | 4 | 4 |  |  |  |
|  |  | Arm 4 | 66 | 4.53 | 0.56 | 3 | 5 |  |  |  |
|  |  | Total | 68 | 4.51 | 0.56 | 3 | 5 |  |  |  |
|  | Learning about treatment options when referral is not possible | Arm 2 | 0 |  |  |  |  | 2.26 | 0.133 |  |
|  |  | Arm 3 | 2 | 4 | 0 | 4 | 4 |  |  |  |
|  |  | Arm 4 | 66 | 4.55 | 0.56 | 3 | 5 |  |  |  |
|  |  | Total | 68 | 4.53 | 0.56 | 3 | 5 |  |  |  |
| Booster session | Overall session | Arm 2 | 56 | 4.46 | 0.60 | 3 | 5 | 8.98 | **0.011** | Arm 2 < Arm 4 |
|  |  | Arm 3 | 19 | 4.32 | 1 | 1 | 5 |  |  |  |
|  |  | Arm 4 | 33 | 4.82 | 0.39 | 4 | 5 |  |  |  |
|  |  | Total | 108 | 4.55 | 0.66 | 1 | 5 |  |  |  |
|  | Exchange of experience with other providers | Arm 2 | 56 | 4.30 | 0.74 | 2 | 5 | 2.04 | 0.361 |  |
|  |  | Arm 3 | 19 | 4.47 | 0.61 | 3 | 5 |  |  |  |
|  |  | Arm 4 | 33 | 4.55 | 0.51 | 4 | 5 |  |  |  |
|  |  | Total | 108 | 4.41 | 0.66 | 2 | 5 |  |  |  |
|  | Getting practical solutions to problems | Arm 2 | 55 | 4.36 | 0.68 | 3 | 5 | 3.70 | 0.157 |  |
|  |  | Arm 3 | 19 | 4.32 | 0.75 | 3 | 5 |  |  |  |
|  |  | Arm 4 | 33 | 4.64 | 0.49 | 4 | 5 |  |  |  |
|  |  | Total | 107 | 4.44 | 0.65 | 3 | 5 |  |  |  |

*in Colombia, some providers from Arm 3 could only attend the training session scheduled for Arm 4

*Table S8:* Summary of open answers to post-training questionnaire; comments regarding training and suggestions for improvements

| **Short training (Arm 2 and 3)** | **Long training Arm 4)** |
| --- | --- |
| *Colombia* |  |
| Content   - Present how to deal with difficult patients - More feedback on exercises   Logistics   - Location of training should be closer to the place of work - Training venue could be more comfortable - Improve punctuality and clarity of timetable | Content:   - More videos and examples could be included - Provide more options for patients referral - Have more time (for each topic)   Logistics:   - Include breakfast |
| *Mexico* |  |
| Content:   - Compliments on the training, not much to add or improve - Role plays are helpful - Include videos that could be shown also to patients - More realistic examples of patients in the videos - More examples and exercises, more detailed information - More time for the training - More information on Mexican statistics   Logistics:   - Include coffee and cake | Content:   - Session 1: clear presentation - Could present more clinical cases and how to deal with them in practice - Some more time would be useful - Session 2: at times too much information, too repetitive and tedious – could be shorter, with less examples and role-play - Having contact person in case of doubts when implementing in practice - More information on complications of alcohol dependence, how to approach a resistant patient   Logistics:   - Include snack   Other:   - Include other providers in the training |
| *Peru* |  |
| Content:   - Compliments on the training - Relevant and important (although often forgotten) topic of the training - More practice (also in front of other providers), individualized feedback - More videos, videos reflecting more closely the reality of own PHCC - More similar trainings/longer training - More scientific evidence on alcohol abuse in the country   Logistics:   - Previous announcement of the training - Having more dates to choose from (to adapt to schedule)   - Not everyone is finished with their consultations at the same time - Having training scheduled earlier in the day - More suitable venue   Other:   - Involve more other providers - Communicate with managers to facilitate having more time to work with patients - Establish WhatsApp group with participating providers to share ideas | Content:   - Role plays are helpful - More examples and more practice - More videos - Longer training - More similar trainings/longer training   Logistics:   - Previous announcement of the training - Change the scheduling, having more dates to choose from - More suitable venue   Other:   - Appreciation to be able to meet providers who are interested in the topic and speak the same language - Involve more other providers from the centre (make it obligatory) |

*Table S9*: Relationship between implementation factors and outcome; comparison between screeners and non-screeners overall and by country

|  |  | Total | | | | Colombia | | | | Mexico | | | | Peru | | | |
| --- | --- | --- | --- | --- | --- | --- | --- | --- | --- | --- | --- | --- | --- | --- | --- | --- | --- |
|  | SCALA protocol delivery | N | M (SD) / % | M-W U / Chi square | Sig. | N | M (SD) / % | M-W U / Chi square | Sig. | N | M (SD) / % | M-W U / Chi square | Sig. | N | M (SD) / % | M-W U / Chi square | Sig. |
| Arm (short vs. long training) | No | 179 | 43.0% Arm 2. 29.1% Arm 3. 27.9% Arm 4 | 0.869 | 0.648 | 16 | 50.0% Arm 2. 25.0% Arm 3. 25.0% Arm 4 | 0.490 | 0.783 | 66 | 31.8% Arm 2. 40.0% Arm 3. 27.3% Arm 4 | 1.032 | 0.597 | 97 | 49.5% Arm 2. 21.6% Arm 3. 28.9% Arm 4 | 1.013 | 0.603 |
|  | Yes | 173 | 38.2% Arm 2. 31.2% Arm 3. 30.6% Arm 4 |  |  | 51 | 41.2% Arm 2. 33.3% Arm 3. 25.5% Arm 4 |  |  | 73 | 34.2% Arm 2. 32.9% Arm 3. 32.9% Arm 4 |  |  | 49 | 40.8% Arm 2. 26.5% Arm 3. 32.7% Arm 4 |  |  |
| Dose (hours of session participated) | No | 179 | 2.52 (1.18) | 18171.5 | **0.003** | 16 | 2.28 (0.84) | 517.0 | 0.094 | 66 | 2.03 (0.70) | 3016.5 | **0.006** | 97 | 2.89 (1.35) | 2885.0 | **0.016** |
|  | Yes | 173 | 2.82 (1.13) |  |  | 51 | 2.84 (0.98) |  |  | 73 | 2.43 (0.88) |  |  | 49 | 3.39 (1.37) |  |  |
| Dose (Numbers of sessions participated) | No | 179 | 1.36 (0.59) | 18829.5 | **<0.001** | 16 | 1.25 (0.45) | 546.0 | **0.019** | 66 | 1.26 (0.44) | 2936.0 | **0.008** | 97 | 1.44 (0.68) | 2885.0 | **0.016** |
|  | Yes | 173 | 1.58 (0.60) |  |  | 51 | 1.59 (0.50) |  |  | 73 | 1.51 (0.48) |  |  | 49 | 1.69 (0.68) |  |  |
| Participation in 1st training * | No | 179 | 86.6% attending T1 13.4% not attending T1 | 0.274 | 0.600 | 16 | 62.5% attending T1 37.5% not attending T1 | 6.808 | **0.009** | 66 | 75.8% attending T1 24.2% not attending T1 | 1.864 | 0.172 | 97 | 97.9% attending T1 2.1% not attending T1 | 3.075 | 0.079 |
|  | Yes | 173 | 88.4% attending T1 11.6% not attending T1 |  |  | 51 | 90.2% attending T1 9.8% not attending T1 |  |  | 73 | 84.9% attending T1 15.1% not attending T1 |  |  | 49 | 91.8% attending T1 8.2% not attending T1 |  |  |
| Participation in 2nd training** | No | 46 | 71.7% attending T2 28.3% not attending T2 | 4.498 | **0.034** |  |  |  |  | 18 | 88.9% attending T2 11.1% not attending T2 | 0.092 | 0.762 | 28 | 60.7% attending T2 39.3% not attending T2 | 3.509 | 0.061 |
|  | Yes | 40 | 90.0% attending T2 10.0% not attending T2 |  |  |  |  |  |  | 24 | 91.7% attending T2 8.3% not attending T2 |  |  | 16 | 87.5% attending T2 12.5% not attending T2 |  |  |
| Participation in booster session* | No | 179 | 30.7% attending B 69.3% not attending B | 12.443 | **<0.001** | 16 | 62.5% attending 37.5% not attending B | 0.207 | 0.649 | 66 | 25.8% attending 74.2% not attending B | 1.577 | 0.209 | 97 | 28.9% attending 71.1% not attending B | 5.743 | **0.017** |
|  | Yes | 173 | 49.1% attending B 50.9% not attending B |  |  | 51 | 68.6% attending B 31.4% not attending B |  |  | 73 | 35.6% attending B 64.4% not attending B |  |  | 49 | 49.0% attending B 51.0% not attending B |  |  |

* *all providers who attended at least one training (N=352)*

***only providers from Arm 4 in Mexico and Peru who attended at least one training (N=86)*

*Table S10:* Relationship between mechanisms of impact-participant response and outcome; comparison between screeners and non-screeners overall and by country

|  |  | |  | Total | | | | Colombia | | | | Mexico | | | | Peru | | | | |
| --- | --- | --- | --- | --- | --- | --- | --- | --- | --- | --- | --- | --- | --- | --- | --- | --- | --- | --- | --- | --- |
|  |  | SCALA protocol delivery | | N | M (SD) | M-W U | Sig. | N | M (SD) | M-W U | Sig. | N | M (SD) | M-W U | Sig. | N | M (SD) | M-W U | Sig. |  |
| Satisfaction | | | | | | | | | | | | | | | | | | | | |
| Training session 1 | Overall experience with the course | No | | 145 | 4.52 (0.53) | 10050.0 | 0.453 | 7 | 4.14 (0.69) | 92.0 | 0.143 | 49 | 4.71 (0.46) | 1260.0 | 0.068 | 89 | 4.45 (0.52) | 1582.5 | **0.023** |  |
|  |  | Yes | | 145 | 4.57 (0.51) |  |  | 38 | 4.53 (0.51) |  |  | 62 | 4.53 (0.53) |  |  | 45 | 4.67 (0.48) |  |  |  |
|  | Information received before the course | No | | 143 | 4.20 (0.76) | 10086.0 | 0.828 | 7 | 3.71 (0.95) | 94.5 | 0.163 | 49 | 4.33 (0.77) | 1462.0 | 0.829 | 87 | 4.17 (0.72) | 1785.5 | 0.492 |  |
|  |  | Yes | | 143 | 4.25 (0.62) |  |  | 38 | 4.08 (0.54) |  |  | 61 | 4.34 (0.63) |  |  | 44 | 4.27 (0.66) |  |  |  |
|  | Location | No | | 144 | 4.43 (0.70) | 8881.0 | **0.020** | 7 | 3.86 (1.07) | 127.0 | 0.831 | 49 | 4.59 (0.61) | 1264.0 | 0.115 | 88 | 4.39 (0.69) | 1806.5 | 0.358 |  |
|  |  | Yes | | 144 | 4.26 (0.69) |  |  | 38 | 4.00 (0.74) |  |  | 61 | 4.39 (0.69) |  |  | 45 | 4.31 (0.60) |  |  |  |
|  | Venue | No | | 145 | 4.43 (0.63) | 9105.0 | **0.029** | 7 | 3.86 (1.07) | 133.0 | 1.000 | 49 | 4.63 (0.49) | 1297.0 | 0.130 | 89 | 4.37 (0.63) | 1892.0 | 0.564 |  |
|  |  | Yes | | 145 | 4.26 (0.69) |  |  | 38 | 3.95 (0.66) |  |  | 62 | 4.44 (0.64) |  |  | 45 | 4.29 (0.69) |  |  |  |
|  | Duration | No | | 144 | 4.38 (0.62) | 9192.5 | 0.077 | 7 | 4.14 (0.69) | 131.5 | 0.958 | 49 | 4.41 (0.64) | 1317.0 | 0.233 | 88 | 4.38 (0.61) | 1809.0 | 0.485 |  |
|  |  | Yes | | 143 | 4.25 (0.62) |  |  | 38 | 4.16 (0.64) |  |  | 61 | 4.28 (0.61) |  |  | 44 | 4.3 (0.63) |  |  |  |
|  | Trainer | No | | 145 | 4.70 (0.48) | 10347.0 | 0.768 | 7 | 4.57 (0.53) | 128.5 | 0.868 | 49 | 4.84 (0.37) | 1424.0 | 0.414 | 89 | 4.63 (0.51) | 1810.5 | 0.267 |  |
|  |  | Yes | | 145 | 4.72 (0.45) |  |  | 38 | 4.61 (0.50) |  |  | 62 | 4.77 (0.42) |  |  | 45 | 4.73 (0.45) |  |  |  |
|  | My participation | No | | 145 | 4.25 (0.61) | 10344.5 | 0.787 | 7 | 4.14 (0.9) | 128.5 | 0.872 | 49 | 4.39 (0.67) | 1329.5 | 0.210 | 89 | 4.18 (0.53) | 1807.0 | 0.266 |  |
|  |  | Yes | | 145 | 4.23 (0.58) |  |  | 38 | 4.13 (0.58) |  |  | 62 | 4.26 (0.60) |  |  | 45 | 4.29 (0.55) |  |  |  |
|  | Other participants | No | | 141 | 4.30 (0.58) | 8893.5 | 0.077 | 7 | 4.00 (0.82) | 118.0 | 0.579 | 48 | 4.40 (0.71) | 1114.5 | **0.038** | 86 | 4.27 (0.47) | 1834.0 | 0.720 |  |
|  |  | Yes | | 141 | 4.16 (0.62) |  |  | 38 | 4.16 (0.55) |  |  | 59 | 4.12 (0.72) |  |  | 44 | 4.23 (0.52) |  |  |  |
| Training session 2 | Overall experience with the course | No | | 27 | 4.59 (0.50) | 367.5 | 0.487 |  |  |  |  | 9 | 4.56 (0.53) | 43.5 | 0.080 | 18 | 4.61 (0.50) | 109.5 | 0.247 |  |
|  |  | Yes | | 30 | 4.50 (0.51) |  |  |  |  |  |  | 15 | 4.20 (0.41) |  |  | 15 | 4.80 (0.41) |  |  |  |
|  | Information received before the course | No | | 27 | 4.33 (0.62) | 375.5 | 0.600 |  |  |  |  | 9 | 4.56 (0.53) | 53.0 | 0.336 | 18 | 4.22 (0.65) | 133.0 | 0.936 |  |
|  |  | Yes | | 30 | 4.23 (0.68) |  |  |  |  |  |  | 15 | 4.27 (0.70) |  |  | 15 | 4.20 (0.68) |  |  |  |
|  | Location | No | | 27 | 4.41 (0.64) | 352.5 | 0.471 |  |  |  |  | 9 | 4.44 (0.73) | 65.5 | 0.894 | 18 | 4.39 (0.61) | 98.5 | 0.233 |  |
|  |  | Yes | | 29 | 4.52 (0.63) |  |  |  |  |  |  | 15 | 4.40 (0.74) |  |  | 14 | 4.64 (0.50) |  |  |  |
|  | Venue | No | | 27 | 4.44 (0.58) | 371.0 | 0.536 |  |  |  |  | 9 | 4.44 (0.53) | 64.0 | 0.813 | 18 | 4.44 (0.62) | 118.5 | 0.494 |  |
|  |  | Yes | | 30 | 4.53 (0.57) |  |  |  |  |  |  | 15 | 4.47 (0.64) |  |  | 15 | 4.60 (0.51) |  |  |  |
|  | Duration | No | | 27 | 4.30 (0.78) | 353.0 | 0.496 |  |  |  |  | 9 | 4.11 (1.05) | 49.0 | 0.358 | 18 | 4.39 (0.61) | 124.5 | 0.670 |  |
|  |  | Yes | | 29 | 4.10 (0.94) |  |  |  |  |  |  | 14 | 3.71 (1.07) |  |  | 15 | 4.47 (0.64) |  |  |  |
|  | Trainer | No | | 27 | 4.56 (0.51) | 346.5 | 0.263 |  |  |  |  | 9 | 4.67 (0.50) | 63.0 | 0.749 | 18 | 4.50 (0.51) | 94.5 | 0.079 |  |
|  |  | Yes | | 30 | 4.70 (0.47) |  |  |  |  |  |  | 15 | 4.60 (0.51) |  |  | 15 | 4.80 (0.41) |  |  |  |
|  | My participation | No | | 27 | 4.11 (0.64) | 342.0 | 0.260 |  |  |  |  | 9 | 3.89 (0.6) | 54.5 | 0.391 | 18 | 4.22 (0.65) | 109.0 | 0.286 |  |
|  |  | Yes | | 30 | 4.30 (0.65) |  |  |  |  |  |  | 15 | 4.13 (0.74) |  |  | 15 | 4.47 (0.52) |  |  |  |
|  | Other participants | No | | 25 | 4.12 (0.53) | 298.0 | 0.199 |  |  |  |  | 8 | 4.00 (0.53) | 49.5 | 0.456 | 17 | 4.18 (0.53) | 92.0 | 0.200 |  |
|  |  | Yes | | 29 | 4.31 (0.66) |  |  |  |  |  |  | 15 | 4.20 (0.77) |  |  | 14 | 4.43 (0.51) |  |  |  |
| Booster session | Overall experience | No | | 41 | 4.51 (0.78) | 1285.5 | 0.963 | 9 | 4.33 (0.71) | 109 | 0.737 | 5 | 4.60 (0.55) | 30.5 | 0.812 | 27 | 4.56 (0.85) | 320.0 | 0.927 |  |
|  |  | Yes | | 63 | 4.56 (0.59) |  |  | 26 | 4.42 (0.64) |  |  | 13 | 4.62 (0.65) |  |  | 24 | 4.67 (0.48) |  |  |  |
| Practical utility | | | | | | | | | | | | | | | | | | | | |
| Training session 1 | Overall training 1 | No | | 141 | 4.63 (0.55) | 9707.5 | 0.771 | 7 | 4.57 (0.53) | 126.5 | 0.810 | 46 | 4.76 (0.48) | 1123.5 | 0.118 | 88 | 4.57 (0.58) | 1665.5 | 0.071 |  |
|  |  | Yes | | 140 | 4.66 (0.49) |  |  | 38 | 4.61 (0.55) |  |  | 57 | 4.63 (0.49) |  |  | 45 | 4.76 (0.43) |  |  |  |
|  | Information on impact of alcohol and costs of alcohol use | No | | 135 | 4.43 (0.65) | 8867.0 | 0.438 | 7 | 4.71 (0.49) | 101.5 | 0.265 | 41 | 4.59 (0.59) | 1091.5 | 0.754 | 87 | 4.33 (0.68) | 1734.0 | 0.233 |  |
|  |  | Yes | | 138 | 4.51 (0.57) |  |  | 38 | 4.42 (0.64) |  |  | 55 | 4.58 (0.50) |  |  | 45 | 4.49 (0.59) |  |  |  |
|  | Discussion on attitudes to alcohol | No | | 136 | 4.48 (0.61) | 8860.5 | 0.427 | 7 | 4.57 (0.53) | 108.0 | 0.367 | 41 | 4.54 (0.64) | 1027.5 | 0.398 | 88 | 4.44 (0.60) | 1903.0 | 0.856 |  |
|  |  | Yes | | 137 | 4.45 (0.54) |  |  | 38 | 4.37 (0.54) |  |  | 55 | 4.47 (0.54) |  |  | 44 | 4.48 (0.55) |  |  |  |
|  | Phrases to start the discussion with the patient | No | | 134 | 4.49 (0.54) | 9114.5 | 0.909 | 7 | 4.57 (0.53) | 109.5 | 0.390 | 41 | 4.44 (0.63) | 1071 | 0.759 | 86 | 4.5 (0.50) | 1849.0 | 0.631 |  |
|  |  | Yes | | 137 | 4.48 (0.53) |  |  | 38 | 4.39 (0.50) |  |  | 54 | 4.50 (0.54) |  |  | 45 | 4.53 (0.55) |  |  |  |
|  | Presentation of screening criteria for SCALA | No | | 136 | 4.43 (0.58) | 8648.0 | 0.242 | 7 | 4.71 (0.49) | 97.5 | 0.199 | 41 | 4.56 (0.59) | 1033.0 | 0.422 | 88 | 4.35 (0.57) | 1442.0 | **0.007** |  |
|  |  | Yes | | 137 | 4.53 (0.52) |  |  | 38 | 4.45 (0.50) |  |  | 55 | 4.49 (0.54) |  |  | 44 | 4.64 (0.49) |  |  |  |
|  | Role play to practice screening | No | | 135 | 4.45 (0.59) | 8989.0 | 0.571 | 7 | 4.29 (0.76) | 132.0 | 0.971 | 41 | 4.44 (0.67) | 1050.0 | 0.513 | 87 | 4.47 (0.55) | 1712.0 | 0.176 |  |
|  |  | Yes | | 138 | 4.49 (0.58) |  |  | 38 | 4.32 (0.53) |  |  | 55 | 4.53 (0.63) |  |  | 45 | 4.60 (0.54) |  |  |  |
|  | Presentation of steps of brief intervention | No | | 135 | 4.47 (0.60) | 8259.0 | 0.061 | 7 | 4.71 (0.49) | 104.5 | 0.302 | 41 | 4.71 (0.60) | 1085.5 | 0.680 | 87 | 4.34 (0.57) | 1523.5 | **0.018** |  |
|  |  | Yes | | 138 | 4.61 (0.53) |  |  | 38 | 4.50 (0.51) |  |  | 55 | 4.71 (0.50) |  |  | 45 | 4.58 (0.58) |  |  |  |
|  | Role play to practice delivering brief intervention | No | | 135 | 4.46 (0.61) | 9155.5 | 0.782 | 7 | 4.57 (0.79) | 108.0 | 0.382 | 41 | 4.49 (0.71) | 1087.5 | 0.736 | 87 | 4.44 (0.54) | 1731.0 | 0.214 |  |
|  |  | Yes | | 138 | 4.47 (0.63) |  |  | 38 | 4.39 (0.64) |  |  | 55 | 4.45 (0.69) |  |  | 45 | 4.56 (0.55) |  |  |  |
| Training session 2 | Overall training 2 | No | | 25 | 4.64 (0.49) | 263.5 | 0.765 | 0^a^ |  |  |  | 7 | 4.57 (0.53) | 17.5 | 0.298 | 18 | 4.67 (0.49) | 108.0 | 0.189 |  |
|  |  | Yes | | 22 | 4.68 (0.48) |  |  | 0^a^ |  |  |  | 7 | 4.29 (0.49) |  |  | 15 | 4.87 (0.35) |  |  |  |
|  | Role playing delivering brief intervention for alcohol with comorbid depressive symptoms | No | | 34 | 4.74 (0.45) | 493.0 | 0.203 | 3 | 5.00 (0.00) | 6.0 | 0.079 | 12 | 4.75 (0.45) | 37.5 | 0.164 | 19 | 4.68 (0.48) | 126.0 | 0.454 |  |
|  |  | Yes | | 34 | 4.59 (0.50) |  |  | 10 | 4.40 (0.52) |  |  | 9 | 4.44 (0.53) |  |  | 15 | 4.8 (0.41) |  |  |  |
|  | Learning about referral | No | | 34 | 4.56 (0.56) | 514.5 | 0.377 | 3 | 4.67 (0.58) | 7.5 | 0.141 | 12 | 4.50 (0.67) | 33.0 | 0.104 | 19 | 4.58 (0.51) | 101.5 | 0.072 |  |
|  |  | Yes | | 34 | 4.41 (0.66) |  |  | 10 | 4.10 (0.57) |  |  | 9 | 4.00 (0.71) |  |  | 15 | 4.87 (0.35) |  |  |  |
|  | Role playing for referring patients | No | | 34 | 4.59 (0.50) | 513.0 | 0.361 | 3 | 4.67 (0.58) | 8.0 | 0.140 | 12 | 4.50 (0.52) | 33.0 | 0.096 | 19 | 4.63 (0.50) | 109.0 | 0.129 |  |
|  |  | Yes | | 34 | 4.44 (0.61) |  |  | 10 | 4.20 (0.42) |  |  | 9 | 4.00 (0.71) |  |  | 15 | 4.87 (0.35) |  |  |  |
|  | Learning about treatment options when referral is not possible | No | | 34 | 4.53 (0.56) | 578.0 | 1.000 | 3 | 4.67 (0.58) | 8.0 | 0.140 | 12 | 4.33 (0.65) | 43.5 | 0.397 | 19 | 4.63 (0.50) | 90.0 | **0.009** |  |
|  |  | Yes | | 34 | 4.53 (0.56) |  |  | 10 | 4.20 (0.42) |  |  | 9 | 4.11 (0.60) |  |  | 15 | 5.00 (0.00) |  |  |  |
| Booster session | Overall session | No | | 41 | 4.54 (0.60) | 1152.0 | 0.265 | 9 | 4.22 (0.83) | 89.5 | 0.238 | 5 | 4.80 (0.45) | 28.0 | 0.573 | 27 | 4.59 (0.50) | 259.5 | 0.130 |  |
|  |  | Yes | | 63 | 4.65 (0.57) |  |  | 26 | 4.58 (0.58) |  |  | 13 | 4.54 (0.78) |  |  | 24 | 4.79 (0.41) |  |  |  |
|  | Exchange of experience with other providers | No | | 41 | 4.22 (0.61) | 948.5 | **0.011** | 9 | 4.00 (0.87) | 79.5 | 0.119 | 5 | 4.40 (0.89) | 31.5 | 0.910 | 27 | 4.26 (0.45) | 219.0 | **0.020** |  |
|  |  | Yes | | 63 | 4.51 (0.67) |  |  | 26 | 4.46 (0.76) |  |  | 13 | 4.46 (0.78) |  |  | 24 | 4.58 (0.50) |  |  |  |
|  | Practical solutions | No | | 41 | 4.34 (0.62) | 1093 | 0.180 | 9 | 4.22 (0.83) | 107.5 | 0.696 | 5 | 4.20 (0.84) | 28.0 | 0.628 | 27 | 4.41 (0.50) | 221.0 | **0.044** |  |
|  |  | Yes | | 62 | 4.48 (0.67) |  |  | 26 | 4.35 (0.75) |  |  | 13 | 4.38 (0.77) |  |  | 23 | 4.70 (0.47) |  |  |  |

* *all providers who attended at least one training (N=352)*

*Table S11*: Relationship between contextual factors and outcome; comparison between screeners and non-screeners overall and by country

|  |  | Total | | | | Colombia | | | | Mexico | | | | Peru | | | |
| --- | --- | --- | --- | --- | --- | --- | --- | --- | --- | --- | --- | --- | --- | --- | --- | --- | --- |
|  | SCALA protocol delivery | N | M (SD) / % | M-W U / Chi square | Sig. | N | M (SD) | M-W U / Chi square | Sig. | N | M (SD) | M-W U / Chi square | Sig. | N | M (SD) | M-W U / Chi square | Sig. |
| Age | No | 175 | 39.57 (12.44) | 13473 | 0.130 | 14 | 31.36 (7.43) | 328.0 | 0.720 | 64 | 34.72 (13.51) | 2027.0 | 0.280 | 97 | 43.96 (10.41) | 2192.0 | 0.444 |
|  | Yes | 170 | 37.79 (12.5) |  |  | 50 | 32.38 (10.48) |  |  | 71 | 36.38 (12.44) |  |  | 49 | 45.35 (10.99) |  |  |
| Gender | No | 179 | 75.3% female. 24.6% male | 0.337 | 0.562 | 16 | 81.3% female. 18.8% male | 0.304 | 0.581 | 66 | 51.9% female. 40.9% male | 4.975 | **0.026** | 97 | 85.6% female. 14.4% male | 0.091 | 0.763 |
|  | Yes | 173 | 78.0% female. 22.0% male |  |  | 51 | 74.5% female. 25.5% male |  |  | 73 | 76.7% female. 23.3% male |  |  | 49 | 83.7% female. 16.3% male |  |  |
| Profession | No | 178 | 32.0% doctors. 14.6% nurses. 5.6% psychologists. 47.8% others | 19.209 | **<0.001** | 16 | 12.5% doctors. 18.8% nurses. 0% psychologists. 68.8% others | 14.531 | **0.002** | 65 | 52.3% doctors. 10.8% nurses. 7.7% psychologists. 29.2% others | 3.530 | 0.317 | 97 | 21.6% doctors. 16.5% nurses. 5.2% psychologists. 56.7% others | 19.638 | **<0.001** |
|  | Yes | 172 | 45.9% doctors. 12.8% nurses. 13.4% psychologists. 27.9% others |  |  | 51 | 52.9% doctors. 25.5% nurses. 2.0% psychologists. 19.6% others |  |  | 72 | 65.3% doctors. 6.9% nurses. 9.7% psychologists. 18.1% others |  |  | 49 | 10.2% doctors. 8.2% nurses. 30.6% psychologists. 51.0% others |  |  |
